# Intra-residue methyl–methyl correlations for valine and leucine residues in large proteins from a 3D-HMBC-HMQC experiment

**DOI:** 10.1007/s10858-019-00287-9

**Published:** 2019-11-12

**Authors:** Lucas Siemons, Harold W. Mackenzie, Vaibhav Kumar Shukla, D. Flemming Hansen

**Affiliations:** Division of Biosciences, Institute of Structural and Molecular Biology, University College London, London, WC1E 6BT UK

**Keywords:** NMR, Methyl-TROSY, Chemical shift assignment, HMBC, Large proteins

## Abstract

Methyl-TROSY based NMR experiments have over the last two decades become one of the most important means to characterise dynamics and functional mechanisms of large proteins and macromolecular machines in solution. The chemical shift assignment of methyl groups in large proteins is, however, still not trivial and it is typically performed using backbone-dependent experiments in a ‘*divide and conquer*’ approach, mutations, structure-based assignments or a combination of these. Structure-based assignment of methyl groups is an emerging strategy, which reduces the time and cost required as well as providing a method that is independent of a backbone assignment. One crucial step in available structure-based assignment protocols is linking the two prochiral methyl groups of leucine and valine residues. This has previously been achieved by recording NOESY spectra with short mixing times or by comparing NOESY spectra. Herein, we present a method based on through-bond scalar coupling transfers, a 3D-HMBC-HMQC experiment, to link the intra-residue methyl groups of leucine and valine. It is shown that the HMBC-HMQC method has several advantages over solely using NOESY spectra since a unique intra-residue cross-peak is observed. Moreover, overlap in the methyl-TROSY HMQC spectrum can easily be identified with the HMBC-HMQC experiment, thereby removing possible ambiguities in the assignment.

**Electronic supplementary material:**

The online version of this article (10.1007/s10858-019-00287-9) contains supplementary material, which is available to authorized users.

## Introduction

The methyl-bearing residues leucine, isoleucine, and valine are often well dispersed throughout a protein and these residues therefore provide significant coverage of the structure. Using ^13^CH_3_ labelled methyl groups as probes within a uniformly deuterated protein has become an indispensable method to characterise large dynamic systems by Nuclear Magnetic Resonance (NMR) spectroscopy. The three-fold rotational axis in combination with sophisticated pulse sequences, which maintain the sensitivity enhancement afforded by the methyl-TROSY effect, have provided a suite of experiments that allow one to probe the structure and dynamics in a wide variety of systems (Tugarinov et al. 2003; Tugarinov and Kay 2003, 2004; Ruschak and Kay 2010; Hansen and Kay 2011; Hansen et al. 2010).

A central challenge faced is the assignment of resonances in the methyl-TROSY HMQC spectrum. A common method used to achieve this is a ‘*divide and conquer*’ approach where large systems are divided into stable subunits or domains and assigned using standard triple-resonance experiments (Salzmann et al. 2000; Tugarinov et al. 2002) Once the resonances are assigned in each subunit, the methyl resonance assignment is transferred to the full complex. Whilst this approach has proven very successful (Sprangers and Kay 2007; Rosenzweig et al. 2013; Gelis et al. 2007), it can only be applied in cases where the protein can be divided into smaller stable units that are amenable to standard triple-resonance experiments.

Recent structure-based chemical shift assignment methods have recast the problem of methyl resonance assignment as a graph matching problem (Pritišanac et al. 2017; Monneau et al. 2017; Xiao et al. 2015). The aim is to build an experimental graph that represents the connectivity of the methyl resonances in a NOESY spectrum and to overlay this with a methyl–methyl network obtained from a known three-dimensional structure. If a unique overlay between these graphs is found then the assignment can be transferred from the known structure to the NOE graph, providing the desired chemical shift assignment. A significant advantage of these approaches is that, when a representative structure is known, they provide an exact and complete search of the solution space. The structure-based methods provide a significant alternative to previous approaches as they can be performed independently of a backbone assignment and so can be utilised when backbone assignments are impossible or unavailable.

A key part of the structure-based strategies is to reduce the available solution space, which scales approximately with *n*!, where *n* is the number of methyl groups. This is achieved by (*1*) assigning the residue type to each methyl resonance in the methyl-TROSY HMQC spectrum, giving *n*_ile_!(*n*_val_)!(*n*_leu_)! solutions, and (*2*) linking intra-residue valine and leucine methyl resonances into pseudoatoms to further reduce the space to *n*_ile_!(*n*_val_/2)!(*n*_leu_/2)! solutions (Pritišanac et al. 2017). Previously, the resonances of the two prochiral methyl groups of valine and leucine were linked using 3D or 4D NOESY spectra recorded with short mixing times. However, this approach has several shortcomings since it can be challenging to distinguish intra-residue NOEs from inter-residue NOEs.

Below we present a 3D-HMBC-HMQC experiment as an efficient method to link intra-residue methyl groups in valine and leucine residues. To demonstrate the applicability of the method, the experiment is applied to two large systems: the 81 kDa Malate synthase G (MSG), and the 360 kDa α7α7 half-proteasome complex of *T. acidophilum.* Intra-residue correlations are observed for approximately 90% of valine and leucine residues in these two systems.

## Materials and methods

### Density functional theory calculations

**Fig. 1:**
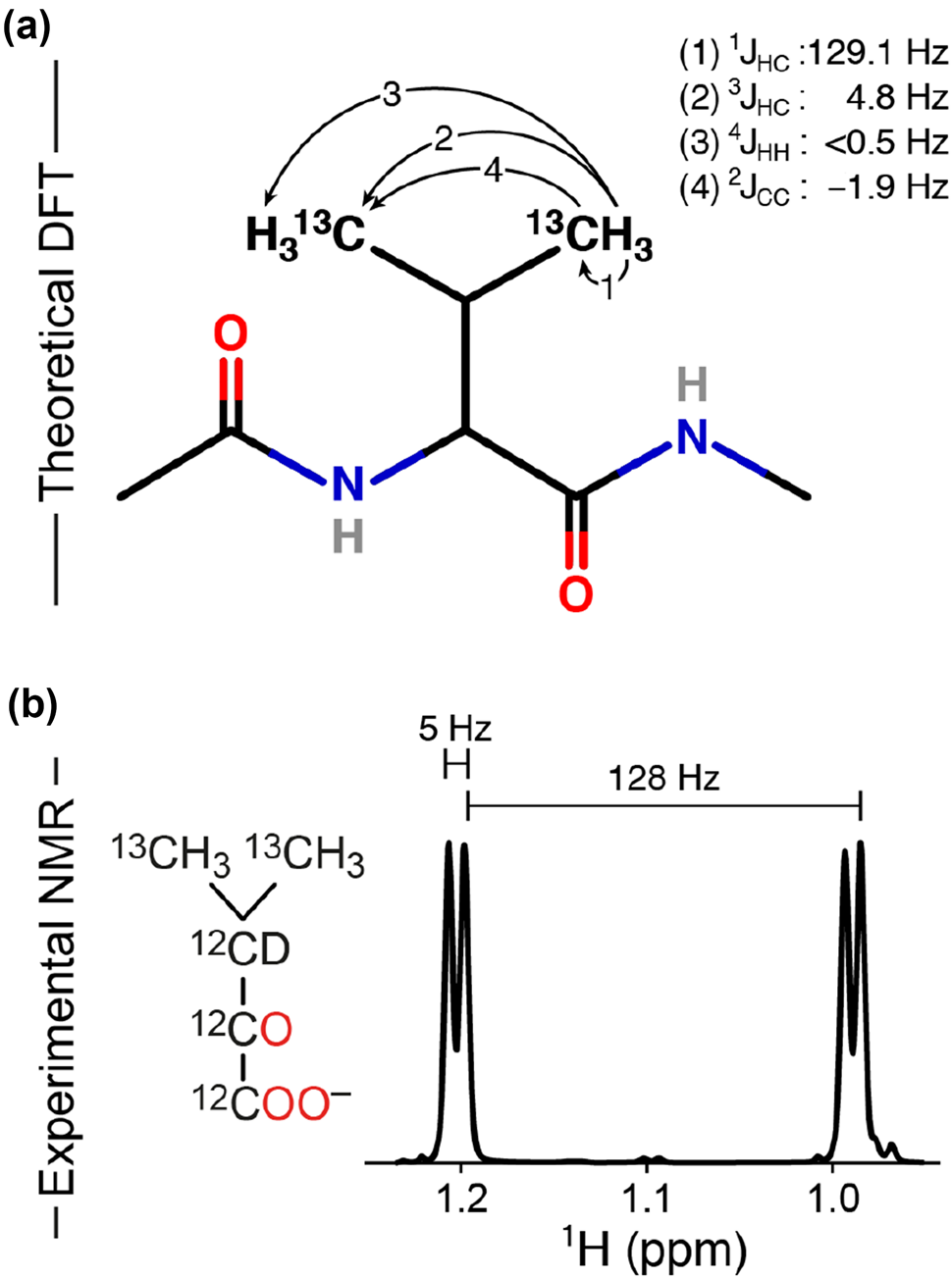
Assessing inter-methyl scalar couplings within a valine side-chain. **a** Scalar couplings between the two methyl groups of the valine side-chain obtained from DFT calculations. **b** A one-dimensional ^1^H NMR (500 MHz) spectrum of {U-[^12^C, ^2^H] [^13^C,^1^H]_2_} α-ketoisovaleric acid. The 128 Hz coupling corresponds to the intra-methyl one-bond ^1^H-^13^C coupling, whereas the ~ 5 Hz coupling is a three-bond inter-methyl ^1^H-^13^C coupling

Scalar coupling constants were obtained from Density Functional Theory (DFT) calculations on the Ac-Val-NMe molecule shown in Fig. 1a using the programme Gaussian 09 (Frisch et al. 2016). Initially a structure optimisation was carried out using the B3LYP functional with the 6-31G* basis set (Ditchfield et al. 1971; Hehre et al. 1972). Subsequently, scalar couplings were calculated using a gauge-independent atomic orbital (GIAO) approach, as implemented in Gaussian with the keyword NMR = SpinSpin.

### Protein expression

Isotopically labelled MSG was produced as described previously (Tugarinov and Kay 2003; Pritchard and Hansen 2019; Korzhnev et al. 2004) with a slight modification. Briefly, the MSG gene, with a C-terminal his_6_ tag, in a kanamycin resistant pET28a vector, was transformed into BL21 (λDE3) *E. coli* cells for protein expression. A single colony was inoculated in 5 mL of LB media supplemented with kanamycin (50 μg mL^−1^) at 37 °C. Once the primary culture reached an OD^600^ between 0.8 and 1.0, it was used to inoculate a 50 ml M9 minimal media culture made with ^2^H_2_O and supplemented with 1 g L^−1^ [^1^H,^15^N]-ammonium chloride and 3 g L^−1^ of [^2^H,^12^C]-glucose as the sole nitrogen and carbon sources, respectively. The pre-culture was used to inoculate 1 L of M9 media and grown at 37 °C to OD^600^ ≈ 0.45. MSG expression was induced for > 16 h with 1 mM IPTG at 21 °C. To achieve the U-[^12^C, ^2^H]-LV-[^13^CH_3_]_2_ methyl labelling {U-[^12^C,^2^H] [^13^CH_3_]_2_} α-ketoisovaleric acid was added one hour prior to induction, while the U-[^12^C, ^2^H]-LV-[^13^CH_3_] labelling scheme was achieved by adding {U-[^12^C,^2^H] [^13^CH_3_]} α-keto-isovalerate.

The cells were lysed by sonication in Lysis buffer (20 mM Tris pH 7.8, 300 mM NaCl, 10 mM 2-mercaptoethanol) supplemented with 10 mg DNase1 (Sigma), 10 mg hen egg lysozyme (Sigma) and 1 complete™ Mini Protease Inhibitor Cocktail tablets (Sigma) per 50 mL lysate. The lysate was pelleted and MSG was purified from the soluble fraction by Ni-NTA affinity chromatography using a HisTrap 5 mL HP column (GE Healthcare), which was pre-equilibrated with Lysis buffer. Protein was eluted from the column using a 10 mM to 250 mM imidazole gradient. The fractions containing MSG were further purified by size exclusion chromatography using a Superdex 200 16/600 gel filtration column (GE Healthcare) (20 mM NaH_2_PO_4_, pH 7.1, 5 mM dithiothreitol).

To produce α-subunit complex (α7α7) of *T. acidophilum* proteasome, the αWT clone, with a N-terminal Histidine tag and a TEV protease site, was transformed into BL21 (λDE3) *E. coli* cells. The protein expression protocol for this α-subunit complex is same as MSG up to induction. The α-subunit complex culture was induced at OD^600^ ≈ 0.9 with 1 mM IPTG at 37 °C for 5 h. The U-[^12^C, ^2^H]-LV-[^13^CH_3_]_2_ methyl labelling scheme was achieved as described above. The cells were lysed by sonication in lysis buffer (50 mM NaH_2_PO_4_ pH 8.0, 0.2 M NaCl, 10 mM imidazole) and purified by Ni-NTA chromatography as before. After purification by Ni-NTA, TEV protease was added and the protein was dialyzed against 2 L of dialysis buffer (50 mM Tris–HCl pH 8.0, 1 mM EDTA, 5 mM 2-mercaptoethanol) overnight at 4 °C. After TEV cleavage the protein was further purified by Ni-NTA chromatography to remove the histidine tag and un-cleaved protein followed by size exclusion chromatography using a Superdex 200 16/600 gel filtration column (GE Healthcare) (50 mM NaH_2_PO_4_ pH 7.5, 0.1 M NaCl).

### NMR spectroscopy

The NMR experiments on MSG were performed on a ~ 400 µM sample in 20 mM Sodium phosphate buffer pH 7.1, 5 mM DTT, 20 mM MgCl_2_, 0.05% NaN_3_ at 37 °C on a Bruker 800 MHz Avance III HD spectrometer equipped with Z-gradient triple-resonance TCI cryoprobe. The 3D-HMBC-HMQC experiment was acquired with 1024, 96, and 64 complex points in the ^1^H, ^13^C_hmqc_, and ^13^C_hmbc_ dimensions with spectral widths of 11161 Hz, 2632 Hz, and 2632 Hz, respectively. Eight scans were collected per increment with a recycling delay of 1 s leading to a total experiment time of 63 h. The transfer time in the HMBC block was 23.5 ms (*n* = 3). The 4D-HMQC-NOESY-HMQC (Vuister et al. 1993; Tugarinov et al. 2005) recorded on MSG was acquired with non-uniform sampling (NUS) over the Nyquist grid consisting of 1024, 64, 72, and 96 complex points with spectral widths of 11161 Hz, 3220 Hz, 3220 Hz, and 2121 Hz, respectively. Four scans were collected per increment with a recycle delay of 1 s and the mixing time was 150 ms. The 4D-HMQC-NOESY-HMQC was recorded using a 1.6% NUS sampling schedule generated with a Poisson Gap distribution (Hyberts et al. 2010). The spectra were reconstructed using an iterative soft thresholding (IST) algorithm (Hyberts et al. 2012) and transformed in nmrPipe (Delaglio et al. 1995). The spectra were processed on the UCL Legion and NMRbox servers (Maciejewski et al. 2017).

The experiments recorded on the α7α7 proteasome were performed on 1.2 mM sample (monomer concentration) in 20 mM potassium phosphate pH 6.8, 50 mM NaCl, 1 mM EDTA, 2 mM DTT, 0.03% NaN_3_ at 50 °C on a Bruker 950 MHz Avance III HD spectrometer equipped with Z-gradient triple-resonance TCI cryoprobe. The 3D-HMBC-HMQC experiment was acquired with 1024, 84, and 48 complex points in the ^1^H, ^13^C_hmqc_, and ^13^C_hmbc_ dimensions, respectively, with spectral widths of 15244 Hz (^1^H) and 3333 Hz (^13^C). 16 scans were collected for each increment with a recycle delay of 1 s for a total experiment time of 80 h.

All spectra were analysed using the CCPN (Vranken et al. 2005) and NMRFAM-Sparky (Lee et al. 2015) software packages. Peak heights used for signal-to-noise calculations were obtained using the inbuilt tools of NMRFAM-Sparky, whereas the noise level was estimated using the inbuilt tool of nmrPipe.

## Results and discussion

Density functional theory (DFT) calculations were carried out on the Ac-Val-NMe molecule, Fig. 1a, in order to assess the possibility of obtaining intra-residue through-bond methyl–methyl correlations in large proteins. This molecule mimics a valine side chain within a protein environment. The calculations show the presence of an inter-methyl three-bond ^13^C-^1^H scalar coupling, ^3^*J*(^1^H^a^,^13^C^b^) = 4.8 Hz, Fig. 1a, which was confirmed experimentally by a 1D ^1^H NMR spectrum of {U-[^12^C,^2^H] [^13^C,^1^H]_2_} α-ketoisovaleric acid, Fig. 1b. The presence of an inter-methyl long-range scalar coupling of ~ 5 Hz, in agreement with previous observations (Reckel et al. 2011; Bax et al. 1994), opens up the possibility for obtaining through-bond methyl–methyl correlations in large proteins for valine and leucine residues.

### The 3D-HMBC-HMQC experiment

**Fig. 2:**
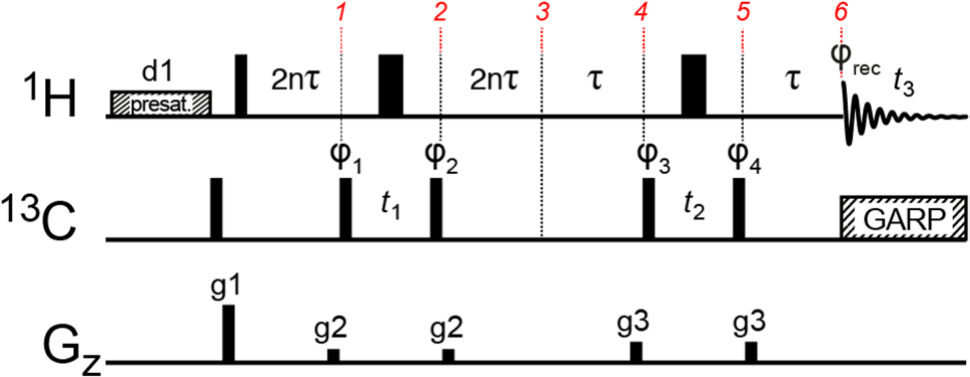
Pulse sequence of the 3D-HMBC-HMQC experiment designed to correlate intra-residue methyl groups of leucine and valine side-chains. The carrier positions are 4.7 and 20 ppm for ^1^H and ^13^C, respectively. Hard 90° (180°) rf-pulses are indicated by narrow (wide) black bars and are applied at the highest available powers. The delay *τ* is set to 3.91 ms and is optimised for 1/(2 × ^1^*J*_CH_), where ^1^*J*_CH_ is 128 Hz. The constant *n* is set to an integer depending on the desired evolution time of the long-range scalar coupling ^3^*J*_CH_. ^13^C decoupling during acquisition is achieved with a 3 kHz GARP4 (Shaka et al. 1985) scheme. Pulses are applied with *x* phase unless stated otherwise. The phase cycle used is φ_1_: *x*, −*x*, φ_3_: 2(*x*), 2(−*x*), φ_rec_: *x*, 2(−*x*), *x*. φ_2_ and φ_4_ are decremented by 90° in accordance with the states-TPPI (Kay et al. 1989) scheme to achieve the required frequency discrimination in F1 and F2, respectively. Gradient pulses of 1 ms are represented by black rectangles and are applied with strengths of g1: 25.1 G cm^−1^, g2: 5.9 G cm^−1^, g3: 9.1 G cm^−1^

The proposed HMBC-HMQC pulse sequence shown in Fig. 2 consists of two back-to-back HMQC elements and is designed to obtain intra-residue methyl–methyl correlations. The first HMQC element (HMBC) (Bax and Summers 1986) is optimised for the three-bond, ^3^*J*_CH_, coupling transfer between the proton of one of the prochiral methyl groups, ^1^H^a^, and the carbon of the other methyl group, ^13^C^b^, while suppressing the one-bond coupling transfer. The second HMQC element is optimised for the standard one-bond ^1^*J*_CH_ coupling transfer.

During the HMBC element, transverse proton magnetisation is allowed to evolve for a period of 2*nτ*, where *n* is an integer and *τ* = 1/(2 × ^1^*J*_CH_). This allows magnetisation to transfer from ^1^H^a^ to ^13^C^b^ while refocusing the one-bond proton–carbon coupling. In the product operator formalism (Sørensen et al. 1984) this results in a density operator at point *1* in Fig. 2 proportional to

1 $$\sigma_{1} \propto - \cos \left( {2\pi \ ^{3}\!\! J n\tau } \right)H_{\text{y}}^{\text{a}} + { \sin }\left( {2\pi\ ^{3}\! Jn\tau } \right) 2H_{\text{x}}^{\text{a}} C_{\text{z}}^{\text{b}}$$

A $90_{{ \pm {\text{x}}}}^{\text{o}}$^13^C pulse generates and selects the inter-methyl multi-quantum (MQ) coherence, $\pm2H_{\text{x}}^{\text{a}} C_{{ {\text{y}}}}^{\text{b}},$ which evolves during *t*_1_ between *1* and *2*. An inversion 180° ^1^H pulse in the middle of the *t*_1_ period refocuses the ^1^H chemical shift. In a subsequent delay of 2*nτ* between *2* and *3* the long-range coupling ^3^*J*_CH_ refocuses and results in a density element at point *3* proportional to

2 $$\sigma_{3} \propto - \sin^{2} \left( {2\pi\ ^{3}\! Jn\tau } \right)H_{\text{y}}^{\text{a}} - \sin \left( {2\pi\ ^{3}\! Jn\tau } \right)\cos \left( {2\pi\ ^{3}\! Jn\tau } \right) 2H_{\text{x}}^{\text{a}} C_{\text{z}}^{\text{b}}$$

The last part of the sequence between point *3* and *6* is a standard HMQC element. It is important to note that the second term in Eq. (2), $2H_{\text{x}}^{\text{a}} C_{\text{z}}^{\text{b}},$ leads to a coherence of the type $4H_{\text{y}}^{\text{a}} C_{\text{z}}^{\text{a}} C_{\text{z}}^{\text{b}}$ at point *4* and is therefore eliminated by the phase cycle of $\phi_{3}.$ The first term in Eq. (2), $H_{\text{y}}^{\text{a}},$ is labelled with the frequency of ^13^C^a^ between *4* and *5* and is finally detected at point *6*, while decoupling ^1^H-^13^C scalar couplings. Fourier transform of the resulting dataset leads to cross-peaks at {ω_1_(^13^C^b^), ω_2_(^13^C^a^), ω_3_(^1^H^a^)}. A key feature of the pulse sequence in Fig. 2 is the absence of ^1^H 90° pulses, which leads to the preservation of the methyl-TROSY effect (Ollerenshaw et al. 2003), thereby increasing the sensitivity of the experiment when applied to large systems.

**Fig. 3:**
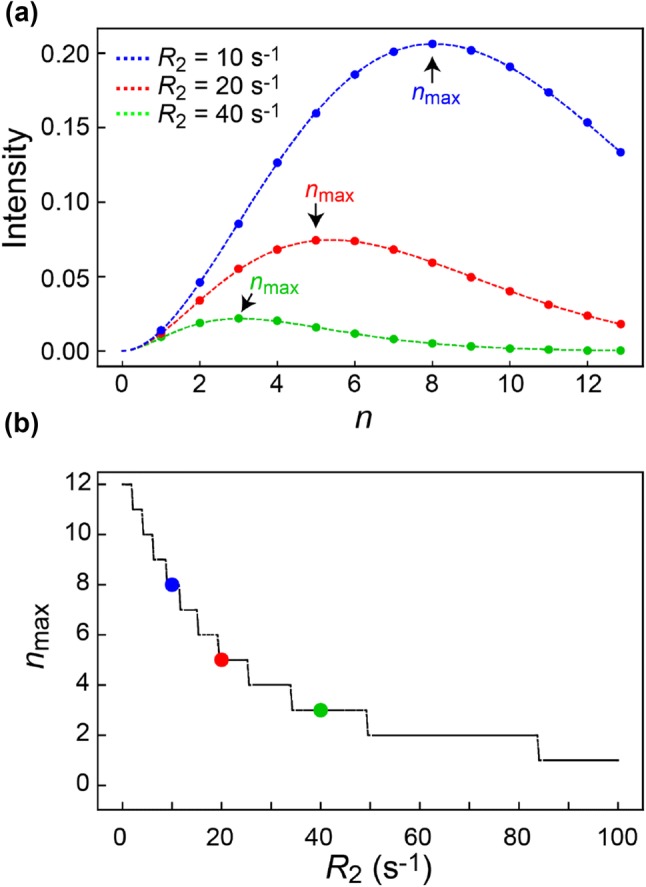
a Plot of the calculated cross-peak intensity as a function of *n* (Fig. 2), for three different transverse relaxation rates, *R*_2_. The intensity on the *y*-axis is normalised to the case where *R*_2_ is 0 s^−1^. **b** Plot of the optimal value of *n, n*_max_ versus *R*_2_. The three cases from *a* are shown with coloured circles

The long-range ^3^*J*_CH_ coupling that gives rise to the desired inter-methyl coherence in Eq (1), $2H_{\text{x}}^{\text{a}} C_{\text{z}}^{\text{b}},$ is only approximately 5 Hz. A compromise between relaxation and scalar-coupling transfer should be considered, since a delay of 1/(2 × ^3^*J*_CH_) = 100 ms, which is required for full transfer, is impractical for large proteins due to relaxation. The dominant relaxation pathway is due to transverse relaxation of ^1^H magnetisation during the 2*t* = 2(2n + 1)*τ* coupling-transfer steps. The signal intensity in the final spectrum is approximately proportional to:

3 $$I ={\text{ sin}}^{ 2} (\pi\ ^{3}\! Jt)\;{\text{exp(}} - 2R_{2} t )$$

where *R*_2_ is the apparent proton transverse relaxation rate. Figure 3 shows the calculated intensity versus the constant *n* for several relaxation rates. The ideal choice for *n* varies from residue to residue depending on the residue-specific transverse relaxation rate, *R*_2_, which in turn depends on the methyl order parameters, *S*_axis_^2^. The HMBC transfer time, 2nτ, can also be optimised using a 2D version of the HMBC-HMQC experiment, where *t*_2_ in Fig. 2 is set to 0 s and *n* is arrayed. Choosing *n* based on the most rigid side chains seems to work well in our hands, since the more flexible side chains generally provide a higher signal-to-noise to begin with.

### Application to the 81 kDa malate synthase G and the 360 kDa α7α7 proteasome

The 3D-HMBC-HMQC approach was initially applied to the 81 kDa MSG, which has 116 leucine and valine residues. The signal-to-noise ratio (S/N) for the 3D-HMBC-HMQC was excellent, with 97% of all possible cross-peaks observed with S/N > 5 (see Fig. S1a), despite the 41 ns effective correlation time (Tugarinov and Kay 2005, 2006). In previous studies 226 leucine and valine methyl-resonances were assigned giving an assignment of 114 residues (L174 and V620 each have one unassigned methyl group), leaving only two residues unassigned. Out of these 226 assigned methyl resonances 9 resonances are marked as ambiguous in the original assignment (Tugarinov and Kay 2003).

**Fig. 4:**
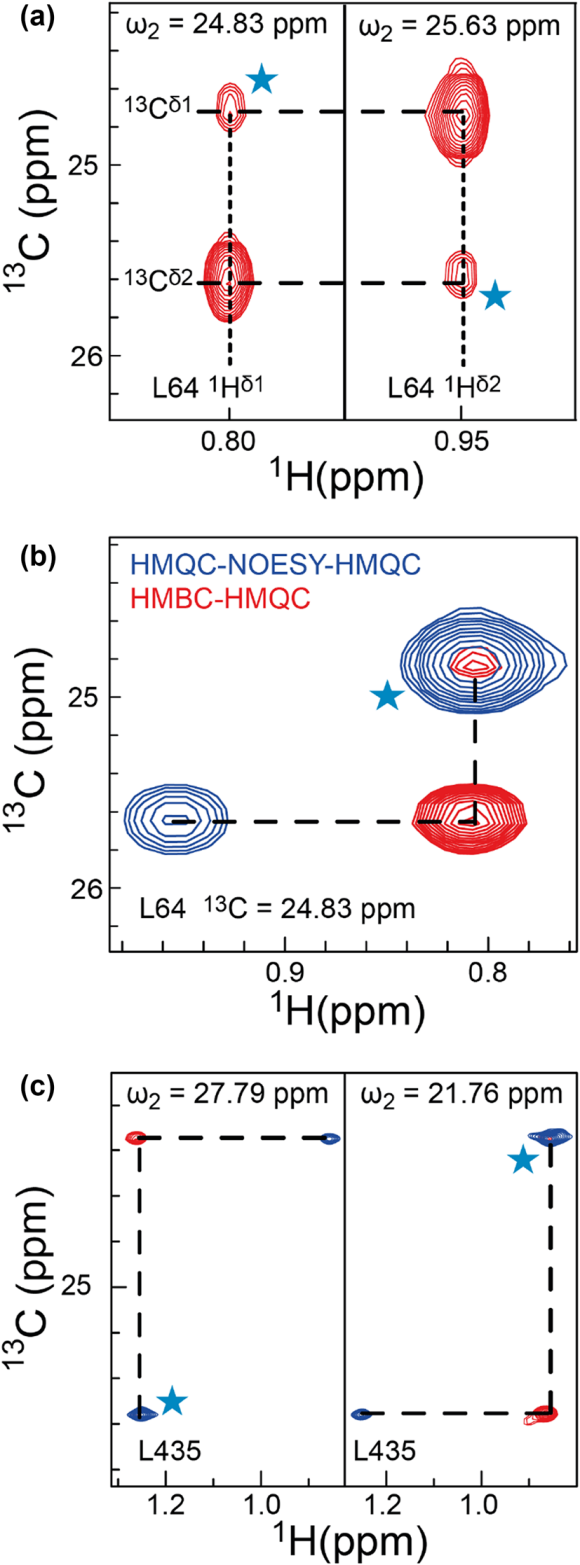
a Two 2D planes of the 3D-HMBC-HMQC experiment on MSG showing the assignments of the two methyl groups of L64. **b** A 2D plane of the 4D-HMQC-NOESY-HMQC and the 3D-HMBC-HMQC spectra for L64 ^13^C^γ1^,^1^H^γ1^. **c** 2D planes of the 4D-HMQC-NOESY-HMQC and the 3D-HMBC-HMQC spectra for linking the two methyl groups of L435. Cyan stars represent diagonal peaks

In the 3D-HMBC-HMQC spectra only two cross peaks should in principle be observed for each valine and leucine residue at (*ω*_1_, *ω*_2_, *ω*_3_) = (^13^C^γ1/δ1^, ^13^C^γ2/δ2^, ^1^H^γ2/δ2^) and at (*ω*_1_, *ω*_2_, *ω*_3_) = (^13^C^γ2/δ2^, ^13^C^γ1/δ1^, ^1^H^γ1/δ1^). This allows the two methyl groups to be correlated using the two ^13^C chemical shifts, Fig. 4a. As also exemplified in Fig. 4a, additional weak diagonal peaks are sometimes observed at (^13^C^γ1/δ1^, ^13^C^γ1/δ1^, ^1^H^γ1/δ1^) and at (^13^C^γ2/δ2^, ^13^C^γ2/δ2^, ^1^H^γ2/δ2^) if the ^1^*J*_CH_ coupling is not completely refocused during the HMBC element. In cases where the methyl-TROSY HMQC spectrum is well-resolved, the 3D-HMBC-HMQC and the 2D-HMQC spectrum contain enough information to link all intra-residue methyl groups of valine and leucine residues. For the 81 kDa MSG, intra-residue methyl–methyl correlations could be confidently assigned for ~ 106 residues using solely the 3D-HMBC-HMQC and the 2D-HMQC experiments.

If the methyl-TROSY HMQC spectrum is crowded it can be challenging to link all the intra-residue methyl cross peaks confidently using only the two ^13^C shifts. For crowded methyl-TROSY HMQC spectra we therefore propose to use the 3D-HMBC-HMQC in combination with a 4D-HMQC-NOESY-HMQC (Vuister et al. 1993; Tugarinov et al. 2005) spectrum recorded with a long mixing time (up to 350 ms depending on size) and utilising a sample where both the *proR* and *proS* methyl groups are labelled with ^13^C and ^1^H in an otherwise deuterated background (U-[^12^C,^2^H]-LV-[^13^C,^1^H]_2_), Fig. 4b, c. The long mixing time guarantees that the intra-residue NOE is observed, which can then be easily identified based on the 3D-HMBC-HMQC, Fig. 4b, c. Using this methodology it is possible to identify intra-residue methyl–methyl cross peaks for 228 methyl resonances in MSG, which resulted in the successful linking of 98.3% of the leucine and valine methyl coherences. Additionally, the 3D-HMBC-HMQC confirms all nine tentative assignments made previously (Tugarinov and Kay 2003) by a backbone dependent assignment and also permits the assignment of the two missing methyl groups, one for L174 (^13^C = 25.70 ppm and ^1^H = 0.95 ppm) and one for V620 (^13^C = 21.45 ppm and ^1^H = 0.82 ppm).

**Fig. 5:**
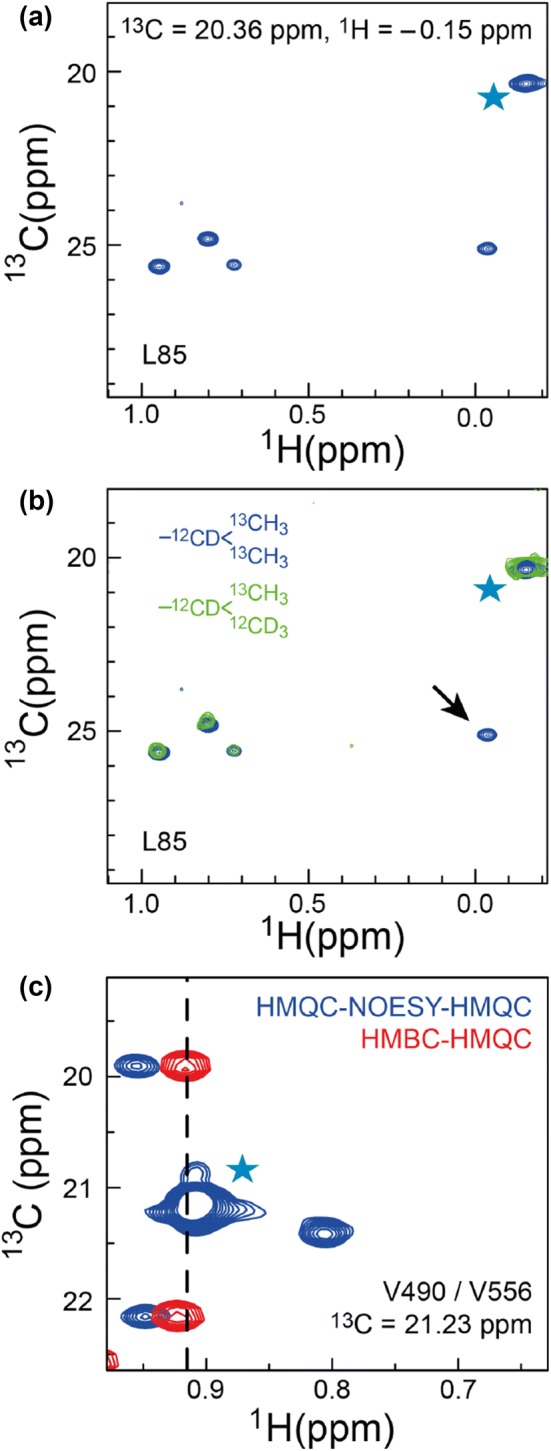
HMBC-HMQC and HMQC-NOESY-HMQC spectra of MSG. **a** A 2D plane extracted from a 4D-HMQC-NOESY-HMQC spectrum focussing on L85. The spectrum was recorded on a doubly U-[^12^C,^2^H]-LV-[^13^C,^1^H]_2_ labelled sample with a mixing time of 150 ms. Multiple cross-peaks with similar intensity are observed, which makes an identification of the intra-residue correlation impossible. **b** An additional 4D-HMQC-NOESY-HMQC spectrum recorded on a singly labelled sample identifies the intra-residue methyl–methyl correlation. The arrow shows the intra-residual methyl–methyl correlation for L85, identified by absence of the peak in the singly methyl labelled sample. **c** Overlap in the 2D methyl-TROSY HMQC spectrum is easily resolved using the 3D-HMBC-HMQC spectrum. Two cross peaks corresponding V490 and V556 are observed. Cyan stars represent diagonal peaks

Indirect methods based on methyl–methyl NOESY spectra, which are independent of the backbone, have traditionally been used to link intra-residue methyl groups of valine and leucine residues in large proteins (Sprangers and Kay 2007). The most commonly used method is to record HMQC-NOESY-HMQC spectra with a short mixing time, in the range of ca. 25–50 ms depending on the size of protein, utilising a U-[^12^C,^2^H]-LV-[^13^C,^1^H]_2_ labelled sample. The aim is to choose a mixing time such that the intra-residue NOE (~ 2.5 Å) can be distinguished from all other inter-residue NOEs. In practice selecting an appropriate mixing time can be challenging without prior knowledge, as exemplified in Fig. 5a for L64 of MSG. This is particularly the case for proteins with varying side-chain dynamics, where the correlation time for the intra-residue methyl–methyl vector can vary greatly throughout the protein and so the observed intra-residue NOEs vary substantially from residue to residue. The scenario is often that for a short NOESY mixing time some valine and leucine residues show no cross peaks, some show a single cross peak, and some may show many cross peaks.

Related to the method above, is a comparison of a HMQC-NOESY-HMQC spectrum recorded on a U-[^12^C,^2^H]-LV-[^13^C,^1^H]_2_ sample with a HMQC-NOESY-HMQC spectrum of a sample with only one of the methyl groups labelled, U-[^12^C,^2^H]-LV-[^13^C,^1^H], Fig. 5b. In the spectrum obtained on the U-[^12^C,^2^H]-LV-[^13^C,^1^H]-sample, one cross peak should be absent compared to the spectra acquired on the U-[^12^C,^2^H]-LV-[^13^C,^1^H]_2_ sample. A key caveat of this approach is that the two labelling schemes lead to different relaxation properties due to the presence/absence of protons on the adjacent methyl, which affects the overall sensitivity. The intra-residue methyl–methyl correlation can be identified confidently using the 3D-HMBC-HMQC spectrum in combination with the HMQC-NOESY-HMQC spectrum, both recorded on a doubly U-[^12^C,^2^H]-LV-[^13^C,^1^H]_2_ labelled sample.

Since the 3D-HMBC-HMQC experiment only utilises scalar coupling transfers it provides additional information compared to the NOESY experiments. First and foremost, only a single cross-peak is observed per methyl group in addition to a potentially easily-identified diagonal peak. This means that if multiple cross-peaks are observed in the 3D-HMBC-HMQC for an apparent single peak in the reference 2D-methyl-HMQC spectrum, then it is a clear indication of overlap in the 2D spectrum. Hence overlapping peaks can be readily identified, as two cross-peaks will be observed, Fig. 5c.

**Fig. 6:**
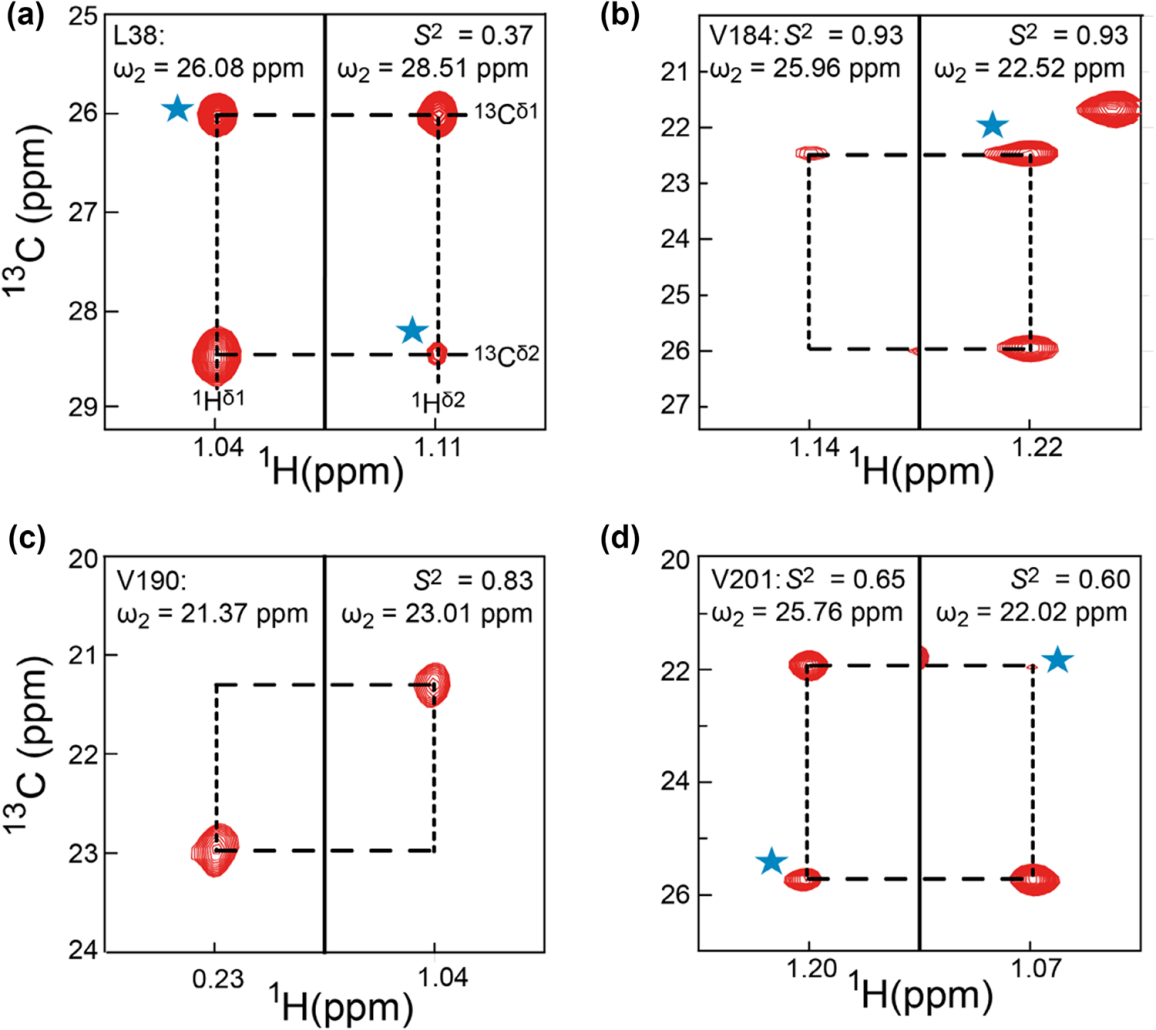
3D-HMBC-HMQC spectra of the 360 kDa α7α7 proteasome to obtain intra-residue methyl–methyl correlations. **a**–**d** are 2D planes of the 3D-HMBC-HMQC spectra for L38, L201, V190, and V184 respectively, which have different order parameters and therefore different internal dynamics. Cyan stars represent diagonal peaks

To demonstrate the utility of the 3D-HMBC-HMQC experiment on larger protein complexes, a 3D-HMBC-HMQC spectrum was recorded on the 360 kDa α7α7 “half-proteasome” from *T. acidophilum*, with an effective rotational correlation time of ~ 120 ns at 50 °C (Tugarinov et al. 2007). Still, good signal-to-noise ratios were generally obtained, Fig. S1b, with 76% of the expected cross-peaks having S/N > 5 and 83% of the valine and leucine residues having at least one HMBC-HMQC cross-peak with S/N > 5. In the α-domain of the proteasome there are 19 leucine and 21 valine residues. In previous studies, 74 leucine and valine methyl-resonances were assigned out of the possible 80 methyl-resonances (Sprangers and Kay 2007). Despite its high molecular weight and slow tumbling, cross peaks with S/N > 3 were observed for 80% of the possible methyl resonances in the 3D-HMBC-HMQC experiment (64 out of 80). Overall, 87.5% of the methyl groups in valine and leucine (35 residues out of 40) side chains were correctly paired. Figure 6a–d demonstrate that despite the size of the α7α7 proteasome the 3D-HMBC-HMQC experiment provides good signal-to-noise across a range of order parameters (Tugarinov et al. 2007).

## Conclusions

In conclusion, we presented a 3D-HMBC-HMQC scalar coupling based approach to link the two prochiral methyl groups in leucine and valine side-chains in large proteins. The method was demonstrated on the 81 kDa Malate synthase G (MSG) and the 360 kDa α7α7 proteasome complex. Only intra-residue correlations are observed since the method utilises scalar-coupling based transfers, which also means that overlap in the 2D-methyl-TROSY HMQC spectrum can be identified by the presence of multiple cross-peaks in the 3D-HMBC-HMQC spectrum. In challenging cases, the presented method can be combined with HMQC-NOESY-HMQC spectra to remove ambiguities in the assignment of intra-residue methyl resonances. Moreover, when methyl resonances are assigned using a mutational approach (Sprangers and Kay 2007; Rosenzweig et al. 2013), the 3D-HMBC-HMQC could also provide valuable insight to confirm the assignments. Finally, the presented approach only relies on a single sample to pair intra-residue methyl resonances and, in favourable cases, it therefore reduces the number of samples required for obtaining the required input for structure-based assignment of methyl NMR resonances.

## Electronic supplementary material

Below is the link to the electronic supplementary material.

Supplementary material 1 (PDF 2114 kb)
