# Supplementary material for: Intra-residue methyl–methyl correlations for valine and leucine residues in large proteins from a 3D-HMBC-HMQC experiment
Source: J Biomol NMR. 2019 Nov 12;73(12):749–57. doi: 10.1007/s10858-019-00287-9 (PMC6875545; doi:10.1007/s10858-019-00287-9)
Supplement: Supplementary file 1 — Supplementary material 1 (PDF 2114 kb) [file 10858_2019_287_MOESM1_ESM.pdf]

Journal of Biomolecular NMR

Intra-residue methyl-methyl correlations for valine and leucine residues in large proteins from a 3D-HMBC-HMQC experiment

--Manuscript Draft--

**Manuscript Number:**	JNMR-D-19-00058R1
**Full Title:**	Intra-residue methyl-methyl correlations for valine and leucine residues in large proteins from a 3D-HMBC-HMQC experiment
**Article Type:**	Article
**Keywords:**	NMR; Methyl-TROSY; Chemical shift assignment; HMBC; large proteins
**Funding Information:**	Biotechnology and Biological Sciences Research Council (BB/R000255/1) — Prof. D. Flemming Hansen<br>Wellcome Trust (101569/z/13/z) — Prof. D. Flemming Hansen<br>Leverhulme Trust (RPG-2016-268) — Prof. D. Flemming Hansen
**Abstract:**	Methyl-TROSY based NMR experiments have over the last two decades become one of the most important means to characterise dynamics and functional mechanisms of large proteins and macromolecular machines in solution. The chemical shift assignment of methyl groups in large proteins is, however, still not trivial and it is typically performed using backbone-dependent experiments in a ‘divide and conquer’ approach, mutations, structure-based assignments or a combination of these. Structure-based assignment of methyl groups is an emerging strategy, which reduces the time and cost required as well as providing a method that is independent of a backbone assignment. One crucial step in available structure-based assignment protocols is linking the two prochiral methyl groups of leucine and valine residues. This has previously been achieved by recording NOESY spectra with short mixing times or by comparing the NOESY spectra. Herein, we present a method based on through-bond scalar coupling transfers, a 3D-HMBC-HMQC experiment, to link the intra-residue methyl groups of leucine and valine. It is shown that the HMBC-HMQC method has several advantages over solely using NOESY spectra since a unique intra-residue cross-peak is observed. Moreover, overlap in the methyl-TROSY HMQC spectrum can easily by identified with the HMBC-HMQC experiment, thereby removing possible ambiguities in the assignment.
**Corresponding Author:**	D. Flemming Hansen, Ph.D.<br>University College London<br>London, UNITED KINGDOM
**Corresponding Author Secondary Information:**	
**Corresponding Author's Institution:**	University College London
**Corresponding Author's Secondary Institution:**	
**First Author:**	Lucas Siemons
**First Author Secondary Information:**	
**Order of Authors:**	Lucas Siemons<br>Harold W Mackenzie<br>Vaibhav Kumar Shukla<br>D. Flemming Hansen, Ph.D.
**Order of Authors Secondary Information:**	
**Author Comments:**	1st of November, 2019

Powered by Editorial Manager® and ProduXion Manager® from Aries Systems Corporation

	Associate Editor, Journal of Biomolecular NMR<br><br>Hi Lewis,<br><br>Please find enclosed our revised manuscript “Intra-residue methyl-methyl correlations for valine and leucine residues in large proteins from a 3D-HMBC-HMQC experiment”<br><br>We are delighted with the favourable responses and positive feedback from the two reviewers as well as the helpful comments to improve our paper. Significant changes to the previous version of the manuscript are highlighted in yellow in the enclosed revised manuscript and attached is also a detailed point-by-point response to the reviewers' comments. Specifically, we have now carried out a more quantitative analysis of the signal-to-noise ratios obtained in the two 3D-HMBC-HMQC spectra recorded for MSG and the a7a7-proteasome (new supporting Figure S1). This will allow the reader to easily judge the range of systems, where the HMBC-HMQC experiment is applicable.<br><br>We believe that the revisions, which were made based on the reviewers' comments, have improved the manuscript and we hope that these changes and additions meet with your approval. Thanks again for your time in consideration of this manuscript – I am looking forward to hearing from.<br><br>All the best<br>Flemming
**Response to Reviewers:**	Please see attached uploaded Word Document under "Response to reviewer comments"

Powered by Editorial Manager® and ProduXion Manager® from Aries Systems Corporation

Manuscript

Click here to access/download;Manuscript;hmbc-hmqc_rev_000.docx

Intra-residue methyl-methyl correlations for valine and leucine residues in large proteins from a 3D-HMBC-HMQC experiment

Lucas Siemons[1,†], Harold W. Mackenzie[1,†], Vaibhav Kumar Shukla[1,*], D. Flemming Hansen[1,*]

[†]) L.S. and H.W.M. contributed equally to this work

1) Institute of Structural and Molecular Biology, Division of Biosciences, University College London, London, United Kingdom, WC1E 6BT

Keywords: NMR, Methyl-TROSY, Chemical shift assignment, HMBC, large proteins.

To whom correspondence should be addressed:

Prof. D. Flemming Hansen; E-mail: d.hansen@ucl.ac.uk or

Dr. Vaibhav Kumar Shukla; E-mail: v.shukla@ucl.ac.uk

Abstract

Methyl-TROSY based NMR experiments have over the last two decades become one of the most important means to characterise dynamics and functional mechanisms of large proteins and macromolecular machines in solution. The chemical shift assignment of methyl groups in large proteins is, however, still not trivial and it is typically performed using backbone-dependent experiments in a '*divide and conquer*' approach, mutations, structure-based assignments or a combination of these. Structure-based assignment of methyl groups is an emerging strategy, which reduces the time and cost required as well as providing a method that is independent of a backbone assignment. One crucial step in available structure-based assignment protocols is linking the two prochiral methyl groups of leucine and valine residues. This has previously been achieved by recording NOESY spectra with short mixing times or by comparing the NOESY spectra. Herein, we present a method based on through-bond scalar coupling transfers, a 3D-HMBC-HMQC experiment, to link the intra-residue methyl groups of leucine and valine. It is shown that the HMBC-HMQC method has several advantages over solely using NOESY spectra since a unique intra-residue cross-peak is observed. Moreover, overlap in the methyl-TROSY HMQC spectrum can easily by identified with the HMBC-HMQC experiment, thereby removing possible ambiguities in the assignment.

Introduction

The methyl-bearing residues leucine, isoleucine, and valine are often well dispersed throughout a protein and these residues therefore provide significant coverage of the structure. Using $^{13}CH_3$ labelled methyl groups as probes within a uniformly deuterated protein has become an indispensable method to characterise large dynamic systems by Nuclear Magnetic Resonance (NMR) spectroscopy. The three-fold rotational axis in combination with sophisticated pulse sequences, which maintain the sensitivity enhancement afforded by the methyl-TROSY effect, have provided a suite of experiments that allow one to probe the structure and dynamics in a wide variety of systems[1–6].

A central challenge faced is the assignment of resonances in the methyl-TROSY HMQC spectrum. A common method used to achieve this is a '*divide and conquer*' approach where large systems are divided into stable subunits or domains and assigned using standard triple-resonance experiments[7,8]. Once the resonances are assigned in each subunit, the methyl resonance assignment is transferred to the full complex. Whilst this approach has proven very successful[9,10,11], it can only be applied in cases where the protein can be divided into smaller stable units that are amenable to standard triple-resonance experiments.

Recent structure-based chemical shift assignment methods have recast the problem of methyl resonance assignment as a graph matching problem[12–14]. The aim is to build an experimental graph that represents the connectivity of the methyl resonances in a NOESY spectrum and to overlay this with a methyl-methyl network obtained from a known three-dimensional structure. If a unique overlay between these graphs is found then the assignment can be transferred from the known structure to the NOE graph, providing the desired chemical shift assignment. A significant advantage of these approaches is that, when a representative structure is known, they provide an exact and complete search of the solution space. The structure-based methods provide a significant alternative to previous approaches as they can be performed independently of a backbone assignment and so can be utilised when backbone assignments are impossible or unavailable.

A key part of the structure-based strategies is to reduce the available solution space, which scales approximately with $n!$, where n is the number of methyl groups. This is achieved by (*1*) assigning the residue type to each methyl resonance in the methyl-TROSY HMQC spectrum, giving $n_{ile}!(n_{val})!(n_{leu})!$ solutions, and (*2*) linking

intra-residue valine and leucine methyl resonances into pseudoatoms to further reduce the space to $n_{ile}!(n_{val}/2)!(n_{leu}/2)!$ solutions[12]. Previously, the resonances of the two prochiral methyl groups of valine and leucine were linked using 3D or 4D NOESY spectra recorded with short mixing times. However, this approach has several shortcomings since it can be challenging to distinguish intra-residue NOEs from inter-residue NOEs.

Below we present a 3D-HMBC-HMQC experiment as an efficient method to link intra-residue methyl groups in valine and leucine residues. To demonstrate the applicability of the method, the experiment is applied to two large systems: the 81 kDa Malate synthase G (MSG), and the 360 kDa α7α7 half-proteasome complex of *T. acidophilum.* Intra-residue correlations are observed for approximately 90% of valine and leucine residues in these two systems.

Materials and methods

Density Functional Theory calculations

Scalar coupling constants were obtained from Density Functional Theory (DFT) calculations on the Ac-Val-NMe molecule shown in Fig. 1a using the programme Gaussian 09[15]. Initially a structure optimisation was carried out using the B3LYP functional with the 6-31G* basis set[16,17]. Subsequently, scalar couplings were calculated using a gauge-independent atomic orbital (GIAO) approach, as implemented in Gaussian with the keyword NMR=spinspin.

Protein expression

Isotopically labelled MSG was produced as described previously[3,18,19] with a slight modification. Briefly, the MSG gene, with a C-terminal his_6 tag, in a kanamycin resistant pET28a vector, was transformed into BL21 (λDE3) *E. coli* cells for protein expression. A single colony was inoculated in 5 ml of LB media supplemented with kanamycin (50 μg/ml) at 37°C. Once the primary culture reached an OD^{600} between 0.8 and 1.0, it was used to inoculate a 50 ml M9 minimal media culture made with $^{2}H_2O$ and supplemented with 1 g L^{-1} [^{1}H,^{15}N]-ammonium chloride and 3 g L^{-1} of [^{2}H,^{12}C]-glucose as the sole nitrogen and carbon sources, respectively. The pre-culture was used to inoculate 1 L of M9 media and grown at 37 °C to OD^{600} ≈ 0.45. MSG expression was induced for >16 hour with 1mM IPTG at 21°C. To achieve the U-[^{12}C, ^{2}H]-LV-[$^{13}CH_3$]$_2$ methyl labelling {U-[^{12}C,^{2}H] [$^{13}CH_3$]$_2$} α-ketoisovaleric acid was added one

hour prior to induction, while the U-[^{12}C, ^{2}H]-LV-[$^{13}CH_3$] labelling scheme was achieved by adding {U-[^{12}C,^{2}H] [$^{13}CH_3$]} α-keto-isovalerate.

The cells were lysed by sonication in Lysis buffer (20 mM Tris pH 7.8, 300 mM NaCl, 10 mM 2-mercaptoethanol) supplemented with 10 mg DNase1 (Sigma), 10 mg hen egg lysozyme (Sigma) and 1 complete™ Mini Protease Inhibitor Cocktail tablets (Sigma) per 50 mL lysate. The lysate was pelleted and MSG was purified from the soluble fraction by Ni-NTA affinity chromatography using a HisTrap 5 mL HP column (GE Healthcare), which was pre-equilibrated with Lysis buffer. Protein was eluted from the column using a 250 mM imidazole gradient. The fractions containing MSG were further purified by size exclusion chromatography using a Superdex 200 16/600 gel filtration column (GE Healthcare) (20mM NaH_2PO_4, pH 7.1, 5mM dithiothreitol).

To produce α-subunit complex (α7α7) of *T. acidophilum* proteasome, the αWT clone, with a N-terminal Histidine tag and a TEV protease site, was transformed into BL21 (λDE3) *E. coli* cells. The protein expression protocol for this α-subunit complex is same as MSG up to induction. The α-subunit complex culture was induced at OD^{600} ≈ 0.9 with 1mM IPTG at 37°C for 5 hours. The U-[^{12}C, ^{2}H]-LV-[$^{13}CH_3$]$_2$ methyl labelling scheme was achieved as described above. The cells were lysed by sonication in lysis buffer (50 mM NaH_2PO_4 pH 8.0, 0.2 M NaCl, 10 mM imidazole) and purified by Ni-NTA chromatography as before. After purification by Ni-NTA, TEV protease was added and the protein was dialyzed against 2 L of dialysis buffer (50 mM Tris-HCl pH 8.0, 1 mM EDTA, 5 mM 2-mercaptoethanol) overnight at 4°C. After TEV cleavage the protein was further purified by Ni-NTA chromatography to remove the histidine tag and un-cleaved protein followed by size exclusion chromatography using a Superdex 200 16/600 gel filtration column (GE Healthcare) (50 mM NaH_2PO_4 pH 7.5, 0.1 M NaCl).

NMR spectroscopy

The NMR experiments on MSG were performed on a ~400 µM sample in 20 mM Sodium phosphate buffer pH 7.1, 5 mM DTT, 20 mM $MgCl_2$, 0.05% NaN_3 at 37 °C on a Bruker 800 MHz Avance HD spectrometer equipped with Z-gradient triple-resonance TCI cryoprobe. The 3D-HMBC-HMQC experiment was acquired with 1024, 96, and 64 complex points in the ^{1}H, $^{13}C_{hmqc}$, and $^{13}C_{hmbc}$ dimensions with spectral widths of 11161 Hz, 2632 Hz, and 2632 Hz, respectively. Eight scans were collected per increment with a recycling delay of 1 s leading to a total experiment time of 63 h.

The transfer time in the HMBC block was 23.5 ms (n = 3). The 4D-HMQC-NOESY-HMQC[20,21] recorded on MSG was acquired with non-uniform sampling (NUS) over the Nyquist grid consisting of 1024, 64, 72, and 96 complex points with spectral widths of 11161 Hz, 3220 Hz, 3220 Hz, and 2121 Hz, respectively. Four scans were collected per increment with a recycle delay of 1 s and the mixing time was 150 ms. The 4D-HMQC-NOESY-HMQC was recorded using a 1.6% NUS sampling schedule generated with a Poisson Gap distribution[22]. The spectra were reconstructed using an iterative soft thresholding (IST) algorithm[23] and transformed in nmrPipe[24]. The spectra were processed on the UCL Legion and NMRbox servers[25].

The experiments recorded on the α7α7 proteasome were performed on 1.2 mM sample (monomer concentration) in 20 mM potasium phosphate pH 6.8, 50 mM NaCl, 1 mM EDTA, 2 mM DTT, 0.03% NaN_3 at 50°C on a Bruker 950 MHz Avance HD spectrometer equipped with Z-gradient triple-resonance TCI cryoprobe. The 3D-HMBC-HMQC experiment was acquired with 1024, 84, and 48 complex points in the ^1H, $^{13}C_{hmqc}$, and $^{13}C_{hmbc}$ dimensions, respectively, with spectral widths of 15244 Hz (^1H) and 3333 Hz (^{13}C). 16 scans were collected for each increment with a recycle delay of 1 s for a total experiment time of 80 h.

All spectra were analysed using the CCPN[26] and NMRFAM-Sparky[27] software packages. Peak heights used for signal-to-noise calculations were obtained using the inbuilt tools of NMRFAM-Sparky, whereas the noise level was estimated using the inbuilt tool of nmrPipe.

Results and Discussion

Density functional theory (DFT) calculations were carried out on the Ac-Val-NMe molecule, Fig. 1a, in order to assess the possibility of obtaining intra-residue through-bond methyl-methyl correlations in large proteins. This molecule mimics a valine side chain within a protein environment. The calculations show the presence of an inter-methyl three-bond ^{13}C-^{1}H scalar coupling, $^{3}J(^{1}H^{a},^{13}C^{b})$ = 4.8 Hz, Fig. 1a, which was confirmed experimentally by a 1D ^{1}H NMR spectrum of {U-[^{12}C,^{2}H] [^{13}C,^{1}H]$_2$} α-ketoisovaleric acid, Fig. 1b. The presence of an inter-methyl long-range scalar coupling of ~5 Hz, in agreement with previous observations[28,29], opens up the possibility for obtaining through-bond methyl-methyl correlations in large proteins for valine and leucine residues.

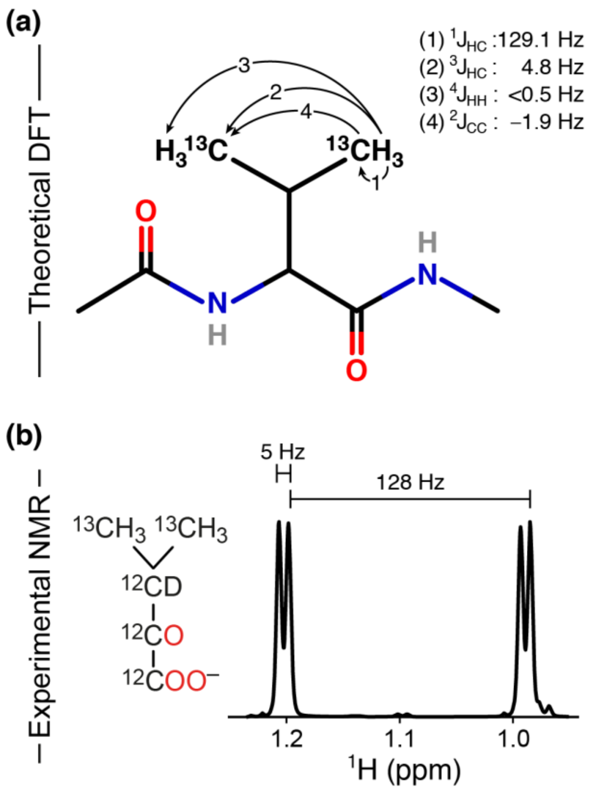

Fig. 1. Assessing inter-methyl scalar couplings within a valine side-chain. (a) Scalar couplings between the two methyl groups of the valine side-chain obtained from DFT calculations. (b) A one-dimensional ^{1}H NMR (500 MHz) spectrum of {U-[^{12}C, ^{2}H] [^{13}C,^{1}H]$_2$} α-ketoisovaleric acid. The 128 Hz coupling corresponds to the intra-methyl one-bond ^{1}H-^{13}C coupling, whereas the ~5 Hz coupling is a three-bond inter-methyl ^{1}H-^{13}C coupling.

The 3D-HMBC-HMQC experiment

The proposed HMBC-HMQC pulse sequence shown in Fig 2 consists of two back-to-back HMQC elements and is designed to obtain intra-residue methyl-methyl correlations. The first HMQC element (HMBC)[30] is optimised for the three-bond, ${}^{3}J_{CH}$, coupling transfer between the proton of one of the prochiral methyl groups, ${}^{1}H^{a}$, and the carbon of the other methyl group, ${}^{13}C^{b}$, while suppressing the one-bond coupling transfer. The second HMQC element is optimised for the standard one-bond ${}^{1}J_{CH}$ coupling transfer.

During the HMBC element, transverse proton magnetisation is allowed to evolve for a period of $2n\tau$, where n is an integer and τ = $1/(2\times{}^{1}J_{CH})$. This allows magnetisation to transfer from ${}^{1}H^{a}$ to ${}^{13}C^{b}$ while refocusing the one-bond proton-carbon coupling. In the product operator formalism[31] this results in a density operator at point *1* in Fig 2 proportional to

$$\sigma_1 \propto -\cos\left(2\pi\ {}^{3}Jn\tau\right)H_{\mathrm{y}}^{\mathrm{a}} + \sin(2\pi\ {}^{3}Jn\tau)\ 2H_{\mathrm{x}}^{\mathrm{a}}C_{\mathrm{z}}^{\mathrm{b}} \qquad (1)$$

A $90^{\mathrm{o}}_{\pm\mathrm{x}}$ ${}^{13}C$ pulse generates and selects the inter-methyl multi-quantum (MQ) coherence, $2H_{\mathrm{x}}^{\mathrm{a}}C_{\pm\mathrm{y}}^{\mathrm{b}}$, which evolves during t_1 between *1* and *2*. An inversion 180° ${}^{1}H$ pulse in the middle of the t_1 period refocuses the ${}^{1}H$ chemical shift. In a subsequent delay of $2n\tau$ between *2* and *3* the long-range coupling ${}^{3}J_{CH}$ refocuses and results in a density element at point *3* proportional to

$$\sigma_3 \propto -\sin^2\left(2\pi\ {}^{3}Jn\tau\right) H_{\mathrm{y}}^{\mathrm{a}} - \sin\left(2\pi\ {}^{3}Jn\tau\right)\cos\left(2\pi\ {}^{3}Jn\tau\right)\ 2H_{\mathrm{x}}^{\mathrm{a}}C_{\mathrm{z}}^{\mathrm{b}} \qquad (2)$$

The last part of the sequence between point *3* and *6* is a standard HMQC element. It is important to note that the second term in Eq. (2), $2H_{\mathrm{x}}^{\mathrm{a}}C_{\mathrm{z}}^{\mathrm{b}}$, leads to a coherence of the type $4H_{\mathrm{y}}^{\mathrm{a}}C_{\mathrm{z}}^{\mathrm{a}}C_{\mathrm{z}}^{\mathrm{b}}$ at point *4* and is therefore eliminated by the phase cycle of ϕ_3. The first term in Eq. (2), $H_{\mathrm{y}}^{\mathrm{a}}$, is labelled with the frequency of ${}^{13}C^{a}$ between *4* and *5* and is finally detected at point *6*, while decoupling ${}^{1}H$-${}^{13}C$ scalar couplings. Fourier transform of the resulting dataset leads to cross-peaks at {$\omega_1({}^{13}C^{b})$, $\omega_2({}^{13}C^{a})$, $\omega_3({}^{1}H^{a})$}. A key feature of the pulse sequence in Fig 2 is the absence of ${}^{1}H$ 90° pulses, which leads to the

preservation of the methyl-TROSY effect[32], thereby increasing the sensitivity of the experiment when applied to large systems.

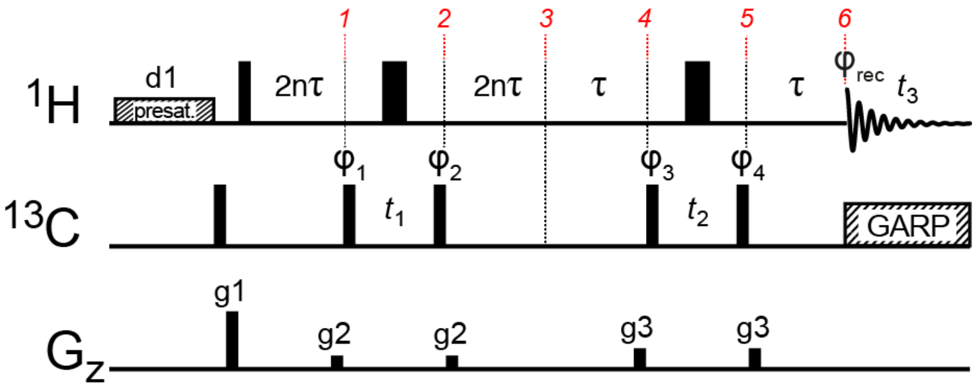

Fig. 2. Pulse sequence of the 3D-HMBC-HMQC experiment designed to correlate intra-residue methyl groups of leucine and valine side-chains. The carrier positions are 4.7 and 20 ppm for ^{1}H and ^{13}C, respectively. Hard 90° (180°) rf-pulses are indicated by narrow (wide) black bars and are applied at the highest available powers. The delay τ is set to 3.91 ms and is optimised for 1/(2 × $^1J_{CH}$), where $^1J_{CH}$ is 128 Hz. The constant n is set to an integer depending on the desired evolution time of the long-range scalar coupling $^3J_{CH}$. ^{13}C decoupling during acquisition is achieved with a 3 kHz GARP4[33] scheme. Pulses are applied with x phase unless stated otherwise. The phase cycle used is φ_1: x, -x, φ_3: 2(x), 2(-x), φ_{rec}: x, 2(-x), x. φ_2 and φ_4 are decremented by 90° in accordance with the states-TPPI[34] scheme to achieve the required frequency discrimination in F1 and F2, respectively. Gradient pulses of 1 ms are represented by black rectangles and are applied with strengths of g1: 25.1 G/cm, g2: 5.9 G/cm, g3: 9.1 G/cm.

The long-range $^3J_{CH}$ coupling that gives rise to the desired inter-methyl coherence in Eq (1), $2H_x^a C_z^b$, is only approximately 5 Hz. A compromise between relaxation and scalar-coupling transfer should be considered, since a delay of 1/(2 × $^3J_{CH}$) = 100 ms, which is required for full transfer, is impractical for large proteins due to relaxation. The dominant relaxation pathway is due to transverse relaxation of ^{1}H magnetisation during the $2t$ = 2(2n+1)τ coupling-transfer steps. The signal intensity in the final spectrum is approximately proportional to:

$$I = \sin^2(\pi\ ^3Jt)\exp(-2R_2t) \quad (3)$$

where R_2 is the apparent proton transverse relaxation rate. Fig. 3 shows the calculated intensity *vs*. the constant n for several relaxation rates. The ideal choice for n varies from residue to residue depending on the residue-specific transverse relaxation rate, R_2, which in turn depends on the methyl order parameters, S^2_{axis}. The HMBC transfer time, 2nτ, can also be optimised using a 2D version of the HMBC-HMQC experiment, where t_2 in Fig. 2 is set to 0 s and n is arrayed. Choosing n based on the most rigid side chains seems to work well in our hands, since the more flexible side chains generally provide a higher signal-to-noise to begin with.

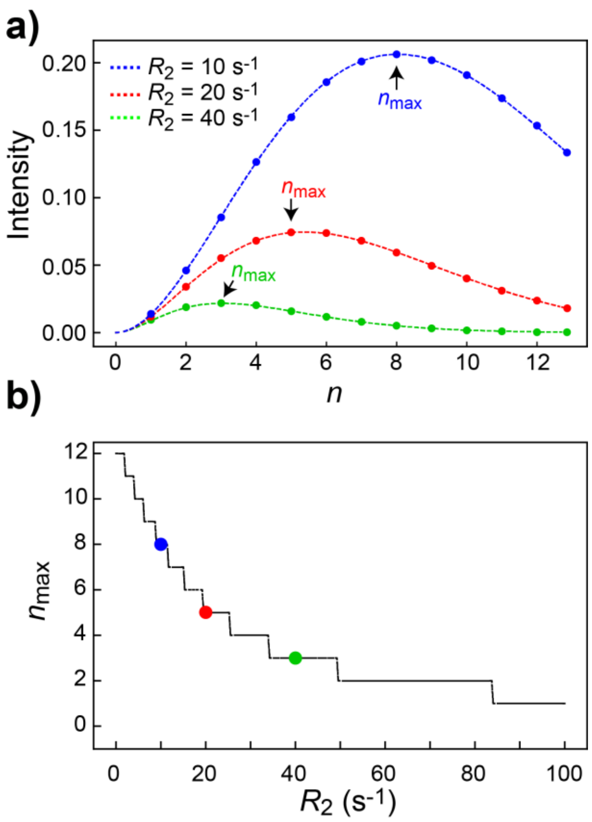

Fig. 3. (a) Plot of the calculated cross-peak intensity as a function of n (Fig 2), for three different transverse relaxation rates, R_2. The intensity on the y-axis is normalised to the case where R_2 is 0 s^{-1}. (b) Plot of the optimal value of n, n_{max} *vs.* R_2. The three cases from *a* are shown with coloured circles.

Application to the 81 kDa Malate Synthase G and the 360 kDa α7α7 proteasome

The 3D-HMBC-HMQC approach was initially applied to the 81 kDa MSG, which has 116 leucine and valine residues. The signal-to-noise ratio (S/N) for the 3D-HMBC-HMQC was excellent, with 97% of all possible cross-peaks observed with S/N

$>$ 5 (see Fig S1a), despite the 41 ns effective correlation time[35,36]. In previous studies 226 leucine and valine methyl-resonances were assigned giving an assignment of 114 residues (L174 and V620 each have one unassigned methyl group), leaving only two residues unassigned. Out of these 226 assigned methyl resonances 9 resonances are marked as ambiguous in the original assignment[3].

In the 3D-HMBC-HMQC spectra only two cross peaks should in principle be observed for each valine and leucine residue at (ω_1, ω_2, ω_3) = ($^{13}C^{\gamma1/\delta1}$, $^{13}C^{\gamma2/\delta2}$, $^{1}H^{\gamma2/\delta2}$) and at (ω_1, ω_2, ω_3) = ($^{13}C^{\gamma2/\delta2}$, $^{13}C^{\gamma1/\delta1}$, $^{1}H^{\gamma1/\delta1}$). This allows the two methyl groups to be correlated using the two ^{13}C chemical shifts, Fig. 4a. As also exemplified in Fig. 4a, additional weak diagonal peaks are sometimes observed at ($^{13}C^{\gamma1/\delta1}$, $^{13}C^{\gamma1/\delta1}$, $^{1}H^{\gamma1/\delta1}$) and at ($^{13}C^{\gamma2/\delta2}$, $^{13}C^{\gamma2/\delta2}$, $^{1}H^{\gamma2/\delta2}$) if the $^{1}J_{CH}$ coupling is not completely refocused during the HMBC element. In cases where the methyl-TROSY HMQC spectrum is well-resolved, the 3D-HMBC-HMQC and the 2D-HMQC spectrum contain enough information to link all intra-residue methyl groups of valine and leucine residues. For the 81 kDa MSG, intra-residue methyl-methyl correlations could be assigned confidently for ~106 residues using solely the 3D-HMBC-HMQC and the 2D-HMQC experiments.

If the methyl-TROSY HMQC spectrum is crowded it can be challenging to link all the intra-residue methyl cross peaks confidently using only the two ^{13}C shifts. For crowded methyl-TROSY HMQC spectra we therefore propose to use the 3D-HMBC-HMQC in combination with a 4D-HMQC-NOESY-HMQC[20,21] spectrum recorded with a long mixing time (up to 350 ms depending on size) and utilising a sample where both the *proR* and *proS* methyl groups are labelled with ^{13}C and ^{1}H in an otherwise deuterated background (U-[^{12}C,^{2}H]-LV-[^{13}C,^{1}H]$_2$), Figs. 4b,c. The long mixing time guarantees that the intra-residue NOE is observed, which then easily can be identified based on the 3D-HMBC-HMQC, Figs. 4b,c. Using this methodology it is possible to identify intra-residue methyl-methyl cross peaks for 228 methyl resonances in MSG, which resulted in the successful linking of 98.3% of the leucine and valine methyl coherences. Additionally, the 3D-HMBC-HMQC confirms all nine tentative assignments made previously[3] by a backbone dependent assignment and also permits the assignment of the two missing methyl groups, one for L174 (^{13}C=25.70 ppm and ^{1}H=0.95 ppm) and one for V620 (^{13}C=21.45 ppm and ^{1}H=0.82 ppm).

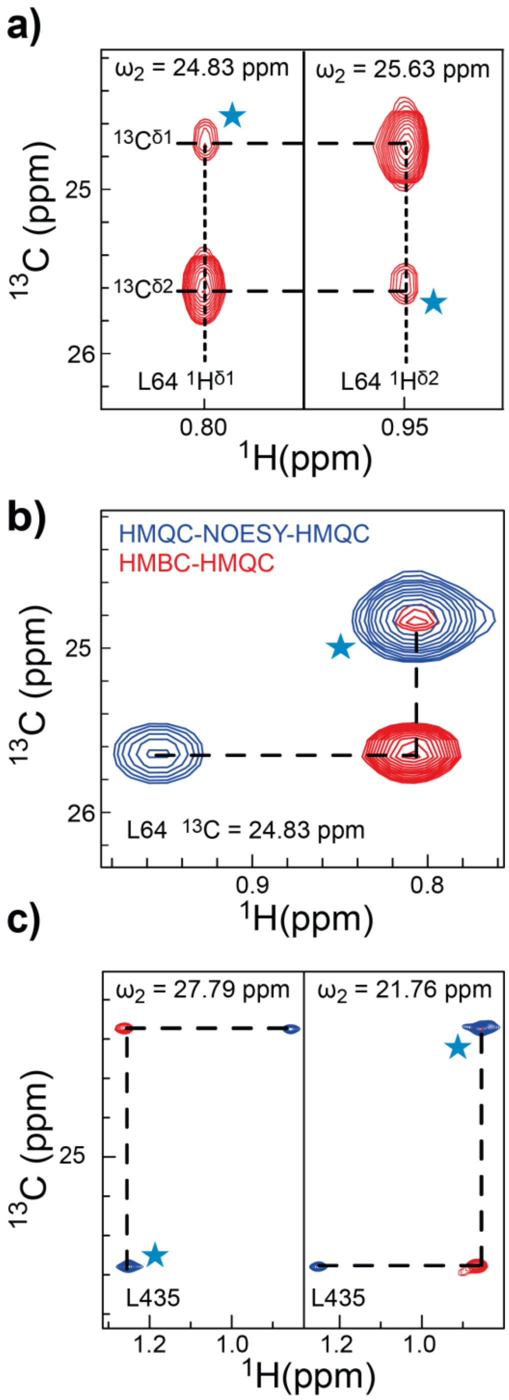

Fig. 4: (a) Two 2D planes of the 3D-HMBC-HMQC experiment on MSG showing the assignments of the two methyl groups of L64 (b) A 2D plane of the 4D-HMQC-NOESY-HMQC and the 3D-HMBC-HMQC spectra for L64 $^{13}C^{\gamma 1}$,$^{1}H^{\gamma 1}$ (c) 2D planes of the 4D-HMQC-NOESY-HMQC and the 3D-HMBC-HMQC spectra for linking the two methyl groups of L435. Cyan stars represent diagonal peaks.

Indirect methods based on methyl-methyl NOESY spectra, which are independent of the backbone, have traditionally been used to link intra-residue methyl groups of valine and leucine residues in large proteins[9]. The most commonly used method is to record HMQC-NOESY-HMQC spectra with a short mixing time, in the range of *ca.* 25-50 ms depending on the size of protein, utilising a U-[^{12}C,^{2}H]-LV-[^{13}C,^{1}H]$_2$ labelled sample. The aim is to choose a mixing time such that the intra-residue NOE (~2.5 Å) can be distinguished from all other inter-residue NOEs. In practice selecting an appropriate mixing time can be challenging without prior knowledge, as exemplified in Fig. 5a for L64 of MSG. This is particularly the case for proteins with varying side-chain dynamics, where the correlation time for the intra-residue methyl-methyl vector can vary greatly throughout the protein and so the observed intra-residue NOE vary substantially from residue to residue. The scenario is often that for a short NOESY mixing time some valine and leucine residues show no cross peaks, some show a single cross peak, and some may show many cross peaks.

Related to the method above, is a comparison of a HMQC-NOESY-HMQC spectrum recorded on a U-[^{12}C,^{2}H]-LV-[^{13}C,^{1}H]$_2$ sample with a HMQC-NOESY-HMQC spectrum of a sample with only one of the methyl groups labelled, U-[^{12}C,^{2}H]-LV-[^{13}C,^{1}H], Fig. 5b. In the spectrum obtained on the U-[^{12}C,^{2}H]-LV-[^{13}C,^{1}H] sample, one cross peak should be absent compared to the spectra acquired on the U-[^{12}C,^{2}H]-LV-[^{13}C,^{1}H]$_2$ sample. A key caveat of this approach is that the two labelling schemes lead to different relaxation properties due to the presence/absence of protons on the adjacent methyl, which affects the overall sensitivity. The intra-residue methyl-methyl correlation can be identified confidently using the 3D-HMBC-HMQC spectrum in combination with the HMQC-NOESY-HMQC spectrum, both recorded on a doubly U-[^{12}C,^{2}H]-LV-[^{13}C,^{1}H]$_2$ labelled sample.

Since the 3D-HMBC-HMQC experiment only utilises scalar coupling transfers it provides additional information compared to the NOESY experiments. First and foremost, only a single cross-peak is observed per methyl group in addition to a potentially easily-identified diagonal peak. This means that if multiple cross-peaks are observed in the 3D-HMBC-HMQC for an apparent single peak in the reference 2D-methyl-HMQC spectrum, then it is a clear indication of overlap in the 2D spectrum. Hence overlapping peaks can be readily identified, as two cross-peaks will be observed, Fig. 5c.

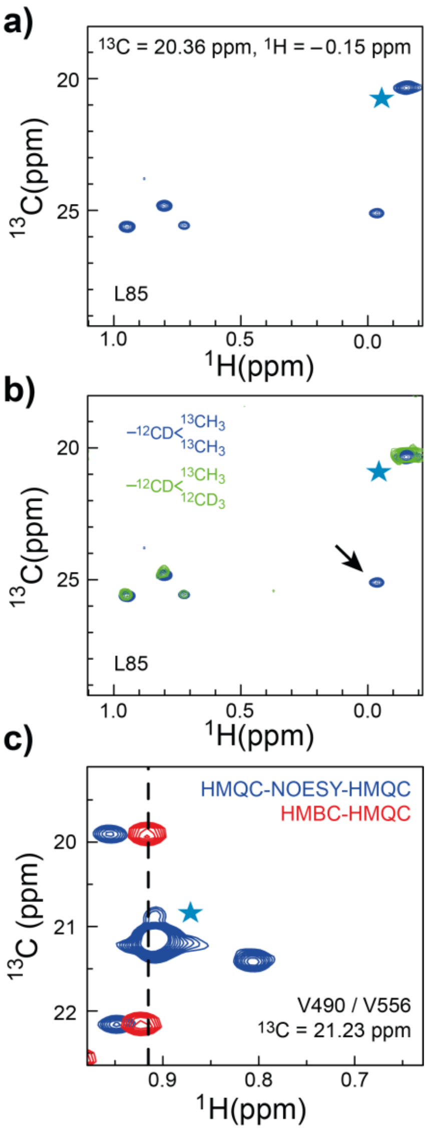

Fig. 5: HMBC-HMQC and HMQC-NOESY-HMQC spectra of MSG. (a) A 2D plane extracted from a 4D-HMQC-NOESY-HMQC spectrum focussing on L85. The spectrum was recorded on a doubly U-[^{12}C,^{2}H]-LV-[^{13}C,^{1}H]$_2$ labelled sample with a mixing time of 150 ms. Multiple cross-peaks with similar intensity are observed, which makes an identification of the intra-residue correlation impossible. (b) An additional 4D-HMQC-NOESY-HMQC spectrum recorded on a singly labelled sample identifies the intra-residue methyl-methyl correlation. The arrow shows the intra-residual methyl-methyl correlation for L85, identified by absence of the peak in the singly methyl labelled sample. (c) Overlap in the 2D methyl-TROSY HMQC spectrum is easily resolved using the 3D-HMBC-HMQC spectrum. Two cross peaks corresponding V490 and V556 are observed. Cyan stars represent diagonal peaks.

To demonstrate the utility of the 3D-HMBC-HMQC experiment on larger protein complexes, a 3D-HMBC-HMQC spectrum was recorded on the 360 kDa α7α7 “half-proteasome” from *T. acidophilum*, with an effective rotational correlation time of

~120 ns[37] at 50 °C. Still, good signal-to-noise ratios were generally obtained, Fig. S1b, with 76% of the expected cross-peaks having S/N > 5 and 83% of the valine and leucine residues having at least one HMBC-HMQC cross-peak with S/N > 5. In the α-domain of the proteasome there are 19 leucine and 21 valine residues. In previous studies, 74 leucine and valine methyl-resonances were assigned out of the possible 80 methyl-resonances[9]. Despite its high molecular weight and slow tumbling, cross peaks with S/N > 3 were observed for 80% of the possible methyl resonances in the 3D-HMBC-HMQC experiment (64 out of 80). Overall, 87.5% of the methyl groups in valine and leucine (35 residues out of 40) side chains were correctly paired. Figs. 5a-d demonstrate that despite the size of the α7α7 proteasome the 3D-HMBC-HMQC experiment provides good signal-to-noise across a range of order parameters[37].

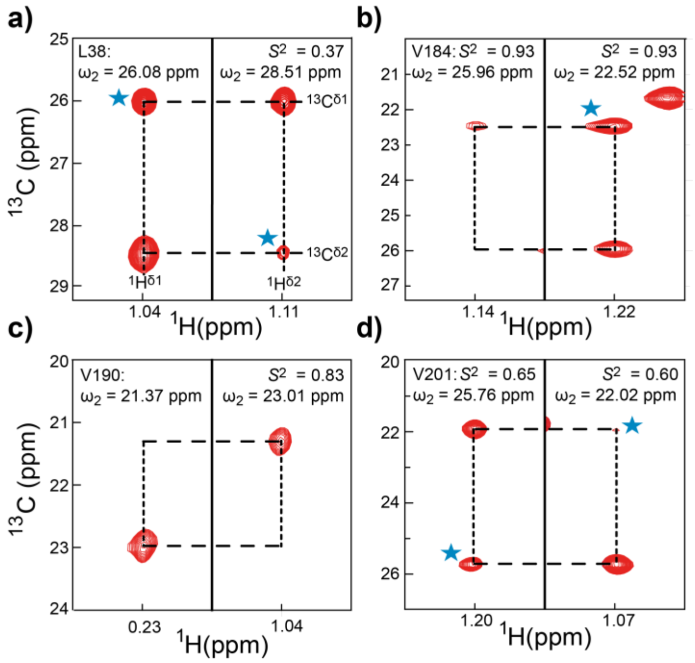

Fig. 6. 3D-HMBC-HMQC spectra of the 360 kDa α7α7 proteasome to obtain intra-residue methyl-methyl correlations. (a), (b), (c), and (d) are 2D planes of the 3D-HMBC-HMQC spectra for L38, L201, V190, and V184 respectively, which have different order parameters and therefore different internal dynamics. Cyan stars represent diagonal peaks.

Conclusions

In conclusion, we presented a 3D-HMBC-HMQC scalar coupling based approach to link the two prochiral methyl groups in leucine and valine side-chains in large proteins. The method was demonstrated on the 81 kDa Malate synthase G (MSG) and the 360 kDa α7α7 proteasome complex. Only intra-residue correlations are

observed since the method utilises scalar-coupling based transfers, which also means that overlap in the 2D-methyl-TROSY HMQC spectrum can be identified by the presence of multiple cross-peaks in the 3D-HMBC-HMQC spectrum. In challenging cases, the presented method can be combined with HMQC-NOESY-HMQC spectra to remove ambiguities in the assignment of intra-residue methyl resonances. Moreover, when methyl resonances are assigned using a mutational approach[9,10], the 3D-HMBC-HMQC could also provide valuable insight to confirm the assignments. Finally, the presented approach only relies on a single sample to pair intra-residue methyl resonances and, in favourable cases, it therefore reduces the number of samples required for obtaining the required input for structure-based assignment of methyl NMR resonances.

Acknowledgements

Prof Lewis E. Kay (University of Toronto) is acknowledged for providing the αWT proteasome clone (α-subunit complex α7α7 from *T. acidophilum*), Dr Angelo M. Figueiredo is acknowledged for help with recording NMR spectra. LS and HWM acknowledge the Wellcome Trust and UCB pharma for PhD studentships, respectively. The BBSRC (BB/R000255/1), Wellcome Trust (ref 101569/z/13/z), and the EPSRC are acknowledged for supporting the ISMB NMR facility at University College London. Access to ultra-high field NMR spectrometers was supported by the Francis Crick Institute through provision of access to the MRC Biomedical NMR Centre. The Francis Crick Institute receives its core funding from Cancer Research UK (FC001029), the UK Medical Research Council (FC001029), and the Wellcome Trust (FC001029). This research is supported by the Leverhulme Trust (RPG-2016-268).

References

1. Tugarinov, V., Hwang, P. M., Ollerenshaw, J. E. & Kay, L. E. Cross-Correlated Relaxation Enhanced 1H−13C NMR Spectroscopy of Methyl Groups in Very High Molecular Weight Proteins and Protein Complexes. *J. Am. Chem. Soc.* **125,** 10420–10428 (2003).
2. Tugarinov, V. & Kay, L. E. An Isotope Labeling Strategy for Methyl TROSY Spectroscopy. *J. Biomol. NMR* **28,** 165–172 (2004).

3. Tugarinov, V. & Kay, L. E. Ile, Leu, and Val methyl assignments of the 723-residue malate synthase G using a new labeling strategy and novel NMR methods. *J. Am. Chem. Soc.* **125,** 13868–78 (2003).
4. Ruschak, A. M. & Kay, L. E. Methyl groups as probes of supra-molecular structure, dynamics and function. *J. Biomol. NMR* **46,** 75–87 (2010).
5. Hansen, D. F. & Kay, L. E. Determining valine side-chain rotamer conformations in proteins from methyl ^{13}C chemical shifts: Application to the 360 kDa half-proteasome. *J. Am. Chem. Soc.* **133,** 8272–8281 (2011).
6. Hansen, D. F., Neudecker, P. & Kay, L. E. Determination of isoleucine side-chain conformations in ground and excited states of proteins from chemical shifts. *J Am Chem Soc* **132,** 7589–7591 (2010).
7. Salzmann, M., Pervushin, K., Wider, G., Senn, H. & Wüthrich, K. NMR Assignment and Secondary Structure Determination of an Octameric 110 kDa Protein Using TROSY in Triple Resonance Experiments. *J. Am. Chem. Soc.* **122,** 7543–7548 (2000).
8. Tugarinov, V., Muhandiram, R., Ayed, A. & Kay, L. E. Four-Dimensional NMR Spectroscopy of a 723-Residue Protein: Chemical Shift Assignments and Secondary Structure of Malate Synthase G. *J. Am. Chem. Soc.* **124,** 10025–10035 (2002).
9. Sprangers, R. & Kay, L. E. Quantitative dynamics and binding studies of the 20S proteasome by NMR. *Nature* **445,** 618–622 (2007).
10. Rosenzweig, R., Moradi, S., Zarrine-Afsar, A., Glover, J. R. & Kay, L. E. Unraveling the mechanism of protein disaggregation through a ClpB-DnaK interaction. *Science* **339,** 1080–1083 (2013).
11. Gelis, I., Bonvin, A. M. J. J., Keramisanou, D., Koukaki, M., Gouridis, G., Karamanou, S., Economou, A. & Kalodimos, C. G. Structural Basis for Signal-Sequence Recognition by the Translocase Motor SecA as Determined by NMR. *Cell* **131,** 756–769 (2007).
12. Pritišanac, I., Degiacomi, M. T., Alderson, T. R., Carneiro, M. G., AB, E., Siegal, G. & Baldwin, A. J. Automatic Assignment of Methyl-NMR Spectra of Supramolecular Machines Using Graph Theory. *J. Am. Chem. Soc.* **139,** 9523–9533 (2017).
13. Monneau, Y. R., Rossi, P., Bhaumik, A., Huang, C., Jiang, Y., Saleh, T., Xie, T., Xing, Q. & Kalodimos, C. G. Automatic methyl assignment in large proteins by

the MAGIC algorithm. *J. Biomol. NMR* **69,** 215–227 (2017).

14. Xiao, Y., Warner, L. R., Latham, M. P., Ahn, N. G. & Pardi, A. Structure-Based Assignment of Ile, Leu, and Val Methyl Groups in the Active and Inactive Forms of the Mitogen-Activated Protein Kinase Extracellular Signal-Regulated Kinase 2. *Biochemistry* **54,** 4307–4319 (2015).
15. Frisch, M. J., Trucks, G. W., Schlegel, H. B., Scuseria, G. E., Robb, M. A., Cheeseman, J. R., Scalmani, G., Barone, V., Mennucci, B., Petersson, G. A., Nakatsuji, H., Caricato, M., Li, X., Hratchian, H. P., Izmaylov, A. F., Bloino, J., Zheng, G., Sonnenberg, J. L., Hada, M., Ehara, M., Toyota, K., Fukuda, R., Hasegawa, J., Ishida, M., Nakajima, T., Honda, Y., Kitao, O., Nakai, H., Vreven, T., Montgomery Jr., J. A., Peralta, J. E., Ogliaro, F., Bearpark, M., Heyd, J. J., Brothers, E., Kudin, K. N., Staroverov, V. N., Kobayashi, R., Normand, J., Raghavachari, K., Rendell, A., Burant, J. C., Iyengar, S. S., Tomasi, J., Cossi, M., Rega, N., Millam, J. M., Klene, M., Knox, J. E., Cross, J. B., Bakken, V., Adamo, C., Jaramillo, J., Gomperts, R., Stratmann, R. E., Yazyev, O., Austin, A. J., Cammi, R., Pomelli, C., Ochterski, J. W., Martin, R. L., Morokuma, K., Zakrzewski, V. G., Voth, G. A., Salvador, P., Dannenberg, J. J., Dapprich, S., Daniels, A. D., Farkas, Ö., Foresman, J. B., Ortiz, J. V, Cioslowski, J. & Fox, D. J. Gaussian 09, Revision A.02. (2016).
16. Ditchfield, R., Hehre, W. J. & Pople, J. A. Self- Consistent Molecular- Orbital Methods. IX. An Extended Gaussian- Type Basis for Molecular- Orbital Studies of Organic Molecules. *J. Chem. Phys.* **54,** 724–728 (1971).
17. Hehre, W. J., Ditchfield, R. & Pople, J. A. Self–Consistent Molecular Orbital Methods. XII. Further Extensions of Gaussian–Type Basis Sets for Use in Molecular Orbital Studies of Organic Molecules. *J. Chem. Phys.* **56,** 2257–2261 (1972).
18. Pritchard, R. B. & Hansen, D. F. Characterising side chains in large proteins by protonless 13C-detected NMR spectroscopy. *Nat. Commun.* **10,** 1747 (2019).
19. Korzhnev, D. M., Kloiber, K., Kanelis, V., Tugarinov, V. & Kay, L. E. Probing Slow Dynamics in High Molecular Weight Proteins by Methyl-TROSY NMR Spectroscopy: Application to a 723-Residue Enzyme. *J. Am. Chem. Soc.* **126,** 3964–3973 (2004).
20. Vuister, G. W., Clore, G. M., Gronenborn, A. M., Powers, R., Garrett, D. S., Tschudin, R. & Bax, A. Increased Resolution and Improved Spectral Quality in

Four-Dimensional $^{13}C/^{13}C$-Separated HMQC-NOESY-HMQC Spectra Using Pulsed Field Gradients. *J. Magn. Reson. Ser. B* **101,** 210–213 (1993).

21. Tugarinov, V., Kay, L. E., Ibraghimov, I. & Orekhov, V. Y. High-Resolution Four-Dimensional ^{1}H–^{13}C NOE Spectroscopy using Methyl-TROSY, Sparse Data Acquisition, and Multidimensional Decomposition. *J. Am. Chem. Soc.* **127,** 2767–2775 (2005).
22. Hyberts, S. G., Takeuchi, K. & Wagner, G. Poisson-Gap Sampling and Forward Maximum Entropy Reconstruction for Enhancing the Resolution and Sensitivity of Protein NMR Data. *J. Am. Chem. Soc.* **132,** 2145–2147 (2010).
23. Hyberts, S. G., Milbradt, A. G., Wagner, A. B., Arthanari, H. & Wagner, G. Application of iterative soft thresholding for fast reconstruction of NMR data non-uniformly sampled with multidimensional Poisson Gap scheduling. *J. Biomol. NMR* **52,** 315–327 (2012).
24. Delaglio, F., Grzesiek, S., Vuister, G. W., Zhu, G., Pfeifer, J. & Bax, A. Nmrpipe - a Multidimensional Spectral Processing System Based on Unix Pipes. *J. Biomol. Nmr* **6,** 277–293 (1995).
25. Maciejewski, M. W., Schuyler, A. D., Gryk, M. R., Moraru, I. I., Romero, P. R., Ulrich, E. L., Eghbalnia, H. R., Livny, M., Delaglio, F. & Hoch, J. C. NMRbox: A Resource for Biomolecular NMR Computation. *Biophys. J.* **112,** 1529–1534 (2017).
26. Vranken, W. F., Boucher, W., Stevens, T. J., Fogh, R. H., Pajon, A., Llinas, M., Ulrich, E. L., Markley, J. L., Ionides, J. & Laue, E. D. The CCPN data model for NMR spectroscopy: development of a software pipeline. *Proteins* **59,** 687–96 (2005).
27. Lee, W., Tonelli, M. & Markley, J. L. NMRFAM-SPARKY: enhanced software for biomolecular NMR spectroscopy. *Bioinformatics* **31,** 1325–1327 (2015).
28. Reckel, S., Gottstein, D., Stehle, J., Löhr, F., Verhoefen, M.-K., Takeda, M., Silvers, R., Kainosho, M., Glaubitz, C., Wachtveitl, J., Bernhard, F., Schwalbe, H., Güntert, P. & Dötsch, V. Solution NMR Structure of Proteorhodopsin. *Angew. Chemie Int. Ed.* **50,** 11942–11946 (2011).
29. Bax, A., Delaglio, F., Grzesiek, S. & Vuister, G. W. Resonance assignment of methionine methyl groups and χ_3 angular information from long-range proton-carbon and carbon-carbon *J* correlation in a calmodulin peptide complex. *J. Biomol. NMR* **4,** 787–797 (1994).

30. Bax, A. & Summers, M. F. Proton and carbon-13 assignments from sensitivity-enhanced detection of heteronuclear multiple-bond connectivity by 2D multiple quantum NMR. *J. Am. Chem. Soc.* **108,** 2093–2094 (1986).
31. Sørensen, O. W., Eich, G. W., Levitt, M. H., Bodenhausen, G. & Ernst, R. R. Product operator formalism for the description of NMR pulse experiments. *Prog. Nucl. Magn. Reson. Spectrosc.* **16,** 163–192 (1984).
32. Ollerenshaw, J. E., Tugarinov, V. & Kay, L. E. Methyl TROSY: explanation and experimental verification. *Magn. Reson. Chem.* **41,** 843–852 (2003).
33. Shaka, A., Barker, P. & Freeman, R. Computer-optimized decoupling scheme for wideband applications and low-level operation. *J. Magn. Reson.* **64,** 547–552 (1985).
34. Kay, L. E., Marion, D. & Bax, A. Practical aspects of 3D heteronuclear NMR of proteins. *J. Magn. Reson.* **84,** 72–84 (1989).
35. Tugarinov, V. & Kay, L. E. Relaxation rates of degenerate ^{1}H transitions in methyl groups of proteins as reporters of side-chain dynamics. *J Am Chem Soc* **128,** 7299–7308 (2006).
36. Tugarinov, V. & Kay, L. E. Quantitative ^{13}C and ^{2}H NMR Relaxation Studies of the 723-Residue Enzyme Malate Synthase G Reveal a Dynamic Binding Interface. *Biochemistry* **44,** 15970–15977 (2005).
37. Tugarinov, V., Sprangers, R. & Kay, L. E. Probing Side-Chain Dynamics in the Proteasome by Relaxation Violated Coherence Transfer NMR Spectroscopy. *J. Am. Chem. Soc.* **129,** 1743–1750 (2007).

Supporting Material

Click here to access/download;Attachment to manuscript (may be uploaded in PDF);Supporting_rev_000.pdf

Supporting Material

Intra-residue methyl-methyl correlations for valine and leucine residues in large proteins from a 3D-HMBC-HMQC experiment

Lucas Siemons, Harold W. Mackenzie, Vaibhav Kumar Shukla, D. Flemming Hansen

Supporting Figures

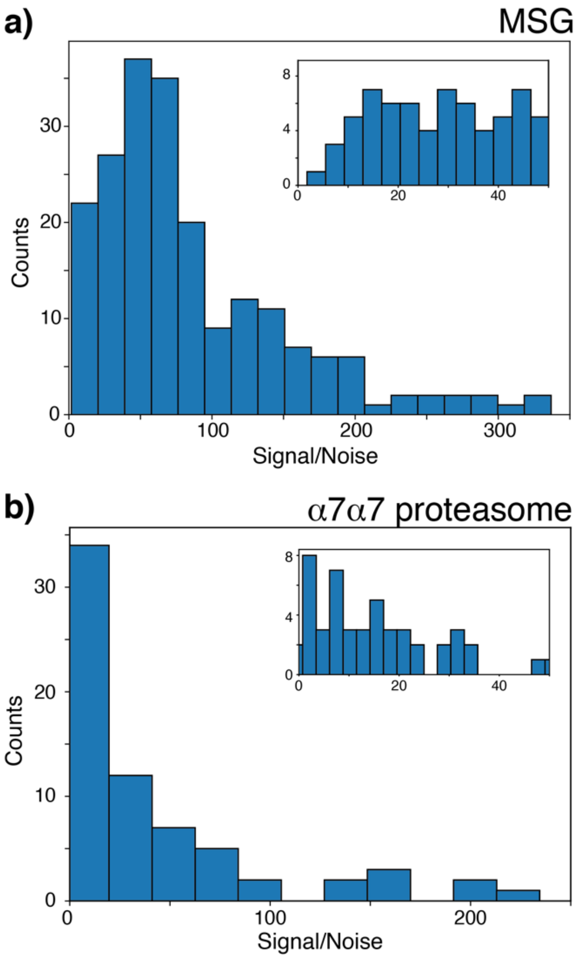

Fig. S1. (a) Histogram of the signal-to-noise ratios obtained in the 3D-HMBC-HMQC spectrum of the 81 kDa Malate Synthase G, recorded at 800 MHz, (37 ºC; $\tau_C \approx 41$ ns). 97% of the possible cross-peaks are observed with signal-to-noise ratios larger than 5. (b) Histogram of the signal-to-noise ratios in the 3D-HMBC-HMQC spectrum of the 360 kDa α7α7 proteasome, recorded at 950 MHz, (50 ºC; $\tau_C \approx 120$ ns). 76% of the expected number of cross-peaks have a signal-to-noise ratio larger than 5.

Pulse sequence – Bruker

```
; 3D HMBC-HMQC sequence for valine/leucine
;
; indirect phase correction 90, 180 in F1 and F2
; includes pre-saturation during d1 at pl19
; HMBC delay set by cnst1
;
; written by Harold Mackenzie on 05/03/2019
;
; F1(H) -> F2(C1, t1) -> F2(C2, t2) -> F1(H, t3)
; 3D (1H, 13C1, 13C2)
;
; *** WARNING: This sequence will not work with ‘getprosol’. Care
; must be taken to ensure that correct pulses have been set prior to
; starting the acquisition ***

;$CLASS=HighRes
;$DIM=3D

#include <Avance.incl>
#include <Grad.incl>
#include <Delay.incl>

;DEFINE PULSES

define pulse pwh
    "pwh=p1"                                 ;1H hard pulse at pl1

define pulse pwc
    "pwc=p2"                                 ;13C hard pulse at pl2

;DEFINE DELAYS

define delay taua
    "taua=1s/(cnst2*2)"                      ;1/2JCH

"d11= 30m"                                   ;Delay for disk
"d12= 2u"                                    ;Delay for power switching
"d16= 200u"                                  ;Delay for grad. recovery

"in0=inf1/2"                                 ;t1 increment
"in10=inf2/2"                                ;t2 increment

"d0=in0/2-pwc-0.63662*pwc"                   ;initial t1 delay
"d10=in10/2-pwc-0.63662*pwc"                 ;initial t2 delay

aqseq 321

;PULSE PROGRAM BEGINS
```

```
1 ze

2 d11 do:f2                                    ;decoupling off
  d12 pl19:f1 pl2:f2                           ;set initial power levels

  d1 cw:f1                                     ;recycle delay
  d12 do:f1                                    ;pre-saturation off

  ;start purge equilibrium 13C magnetisation

  50u UNBLKGRAD                                ;gradient amp on
  (pwc ph10):f2                                ;90x
  2u
  p51:gp1                                      ;cleaning gradient
  d16

  d12 pl1:f1                                   ;power to high (1H)

  ;end purge block, start HMBC

  (pwh ph10):f1
  "DELTA=cnst1*2*taua-0.63662*pwh-2u-p52-d16-0.63662*pwc"
  DELTA
  2u
  p52:gp2
  d16

  (pwc ph1):f2                                 ;start t1 period
  d0
  (pwh*2 ph10):f1
  d0
  (pwc ph2):f2                                 ;end t1 period

  2u
  p52:gp2
  d16
  "DELTA=cnst1*2*taua-0.63662*pwc-2u-p52-d16"
  DELTA

  ;end HMBC, start HMQC

  "DELTA=taua-2u-p53-d16-0.63662*pwc"
  DELTA
  2u
  p53:gp3
  d16

  (pwc ph3):f2                                 ;start t2 period
  d10
  (pwh*2 ph10):f1
```

```
  d10
  (pwc ph4):f2                              ;end t2 period

  2u
  p53:gp3
  d16
  "DELTA=taua-0.63662*pwc-2u-p53-d16-d12-50u"
  DELTA

  d12 pl29:f2                               ;13C power to decouple
  50u BLKGRAD                               ;gradient amp off

  ;end HMQC, start detection of 1H with 13C decoupling

  go=2 ph31 cpd2:f2
  d11 do:f2 mc #0 to 2
    F1PH(calph(ph2,-90), caldel(d0,+in0))
    F2PH(calph(ph4,-90), caldel(d10,+in10))

 exit

;PHASE PROGRAMS

ph1=  0 2
ph2=  0
ph3=  0 0 2 2
ph4=  0
ph31= 0 2 2 0

ph10= 0
ph11= 1
ph12= 2
ph13= 3

;DEFINITIONS

;cnst1    : constant for HMBC delay (3-6)
;cnst2    : JCH (128 Hz)
;p1       : 1H high-power pulse
;plw1     : 1H high-power level
;plw19    : 1H pre-saturation power level
;p2       : 13C high-power pulse
;plw2     : 13C high-power level
;cpd2     : decoupling according to sequence defined by cpdprg2
;pcpd2    : f2 channel - 90 degree pulse for decoupling sequence
;plw29    : 13C decoupling power level

;inf1     : 1/SW = 2 * DW
;inf2     : 1/SW = 2 * DW
;in0      : 1/(2 * SW) = DW
;in10     : 1/(2 * SW) = DW
```

```
;NS       : 4 * n
;DS       : 16
;td1      : number of experiments (13C1)
;td2      : number of experiments (13C2)
;FnMODE   : States-TPPI (F1, F2)

;for z-only gradients:
;gpz1     : 47% (spoil)
;gpz2     : 11%
;gpz3     : 17%

;use gradient files:
;gpnam1   : SMSQ10.100
;gpnam2   : SMSQ10.100
;gpnam3   : SMSQ10.100
```

Response to Reviewers:

Reviewer 1.

The nuances of the NMR experiment and the approach are very well described and illustrated. Overall, this is a very nice contribution that definitely deserves publication in the Journal of Biomolecular NMR after some minor issues are addressed and some copy-editing is performed (the paper is a little bit cryptic in places despite that there are practically no size limitations for Articles in JBNMR).

We are delighted with the favourable comments on our manuscript. Below is a point-by-point response to the specific comments, where the referee's comments are in *italic*, our responses are in black, and new text included in the revised manuscript is highlighted in yellow.

1. *Although HSQC and HMQC experiments can be considered a 'common knowledge' by now and citations of the original publications is optional, the reference to HMBC type of transfer is absolutely central to the work presented and (I think) should be cited. To make life somewhat easier for the authors, here it is:*

   Bax A,Summers M. 1986. Sensitivity- enhanced detection of heteronuclear multiple-bond connectivity by 2D multiple quantum NMR. J Am Chem Soc 108: 2093- 2094.

We have now included a citation to the HMBC experiment, as suggested by the reviewer. For example, in the Results section, page 8,

…The first HMQC element (HMBC)[24] is optimised…

2. *I would not call a NOE mixing time in the range 50-150 ms 'short' (p. 13). Even for the molecules the size of MSG, the intra-residue methyl NOEs can probably be detected with mixing times of about 25-40 ms. This is specified in the paper on methyl assignments of MSG abundantly cited by the authors - please check.*

We thank the reviewer for pointing this out. We have changed the mixing time to the more appropriate range 25-50 ms, page 13,

…with a short mixing time, in the range of ca. 25-50 ms depending…

Minor Issues/Typos/Copy-editing:

a. *'divide-and-conquer' in the abstract should come with quotes and not bold-faced.*

This has been corrected

b. *1st line of page 4: "a pseudo-atoms" should be either "pseudoatoms" or rephrased.*

This has now been corrected:

…into pseudoatoms to further …

c. *1st line of p.7: delete 'Theoretical' and make it "calculations"*

This has now been corrected:

Density functional theory (DFT) calculations…

d. *p.8: "standard HMQC element"*

This has now been corrected

e. *p.9: ".... long range 3JCH coupling that gives ..", " .... the apparent proton transverse relaxation RATE", " ..... the ideal choice for n varies from residue to residue ......"   or rephrase.*

These corrections have now been made

f. *p.10: " ... which in turn depends on ...."*

This has now been corrected

g. *p. 11: " the long mixing time guarantees that "   --- that repeated twice.*

This has now been corrected

h. *The stars in all Figures of the paper are not quite 'cyan' in my version of the printout. It maybe smth is wrong with our cyan cartridge or in the figures themselves --- please check.*

The stars were originally cyan with a black outline. We have now removed the outline and made the stars slightly larger.

i. *p.13, the last sentence of the 2nd paragraph: " Using .... means that the intra-residue ..." --- sounds very awkward -- please rephrase.*

The sentence has now been rephrased, to read,

The intra-residue methyl-methyl correlation can be identified confidently using the 3D-HMBC-HMQC spectrum in combination with the HMQC-NOESY-HMQC spectrum, both recorded on a doubly U-[12C,2H]-LV-[13C,1H]2 labelled sample.

j. *Caption to Fig. 5: Rephrase the first sentence --- it is not clear what (a) and (b) refer to in the 1st sentence. Describe where the arrow in (b) points to.*

The legend to Fig. 5 has now been rephrased, to read,

Fig 5. HMBC-HMQC and HMQC-NOESY-HMQC spectra of MSG. (a) A 2D plane extracted from a 4D-HMQC-NOESY-HMQC spectrum focussing on L85. The spectrum was recorded on a doubly U-[12C,2H]-LV-[13C,1H]2 labelled sample with a mixing time of 150 ms. Multiple cross-peaks with similar intensity are observed, which makes an identification of the intra-residue correlation impossible. (b) An additional 4D-HMQC-NOESY-HMQC spectrum recorded on a singly labelled sample identifies the intra-residue methyl-methyl correlation. The arrow shows the intra-residual methyl-methyl correlation for L85, identified by absence of the peak in the singly methyl labelled sample …

k. *p.15: top line --- rephrase the sentence starting with "As such ..." or eliminate "as such"*

The paragraph that the reviewer is referring to has now been substantially rephrased (see also below and answer to reviewer 2).

… with an effective rotational correlation time of ~120 ns[31] at 50 °C. Still, good signal-to-noise ratios were generally obtained, Fig. S1b, with 76% of the expected cross-peaks having S/N > 5 and 83% of the valine and leucine residues having at least one HMBC-HMQC cross-peak with S/N > 5 …

and

In previous studies, 74 leucine and valine methyl-resonances were assigned out of the possible 80 methyl-resonances[7]. Despite its high molecular weight and slow tumbling, cross peaks with S/N > 3 were observed for 80% of the possible methyl resonances in the 3D-HMBC-HMQC experiment (64 out of 80). Overall, 87.5% of the methyl groups in valine and leucine (35 residues out of 40) side chains were correctly paired …

l. *p.15 Conclusions: the authors probably mean " .... of multiple cross-peaks in the 3D-HMBC-HMQC spectrum". I gather that NOESY can have multiple peaks no matter what ?*

We agree – it should have been '3D-HMBC-HMQC spectrum'. This has now been corrected.

m. *p.15 Conclusions: " provide both the certainty and resolution ..." --- please rephrase.*

The sentence has now been rephrased, to read,

In challenging cases, the presented method can be combined with HMQC-NOESY-HMQC spectra to remove the chance of any ambiguity in the assignment of intra-residue methyl resonances.

n. *p.15 last line, Conclusions : "resonances" in plural*

The last line has now been rephrased, to read,

…structure-based assignment of methyl NMR resonances.

o. *The references do not seem to be formatted in the text and the Bibliography according to the rules of JBNMR -- please check.*

It is our understanding, from the 'Instructions for Authors' that the reference style changed in 2017 and it is now the same as Nature Structural and Molecular Biology.

Reviewer 2.

Below is a point-by-point response to the specific comments, where the referee's comments are in *italic*, our responses are in black, and new text included in the revised manuscript is highlighted in yellow.

1. *The authors described that the HMBC transfer time (n) should be optimized considering the relaxation properties of the methyl protons based on the simulations. Is it possible to experimentally optimize the HMBC transfer time (n) with the 2D versions of HMBC-HMQC spectra?*

Indeed, for both MSG and the half-proteasome, we initially optimised the transfer time using a 2D version of the HMBC-HMQC experiment, with n=2, 3, 4. We have now briefly described this procedure in the manuscript on page 10,

The HMBC transfer time, $2n\tau$, can also be optimised using a 2D version of the HMBC-HMQC experiment, where t_2 in Fig. 2 is set to 0 s and *n* is arrayed. …

2. *One may wonder whether the HMBC-HMQC is applicable to his/her proteins of interest. It is helpful for readership to describe the signal-to-noise ratio of the HMBC-HMQC spectra and the transverse relaxation rates (R_2) of the methyl resonances for MSG and the half-proteasome.*

We had previously, in Fig. 6, given the order parameters in order to show that the 3D-HMBC-HMQC method works well for both rigid and flexible side chains of the 360 kDa 'half' proteasome. We have carried out a more quantitative analysis of the obtained signal-to-noise ratios. Specifically, we have quantified the signal-to-noise for the expected cross-peaks in the obtained 3D-HMBC-HMQC spectra for MSG and the half-proteasome and generated a new figure in supporting material to show this (Figure S1). Instead of providing R_2 rates, we have focussed on the size and effective rotational correlation times, which we believe will allow one to easily judge the range of systems, where the 3D-HMBC-HMQC experiment is applicable.

In the main text we have added the following for MSG:
The signal-to-noise ratio (S/N) for the 3D-HMBC-HMQC was excellent, with 97% of all possible cross-peaks observed with S/N > 5 (see Fig S1a), despite the 41 ns effective correlation time[29,30].

and for the half-proteasome:
… with an effective rotational correlation time of ~120 ns[31] at 50 °C. Still, good signal-to-noise ratios were generally obtained, Fig. S1b, with 85% of the expected cross-peaks having S/N > 5 and 92% of the valine and leucine residues having at least one HMBC-HMQC cross-peak with S/N > 5.

3. *In P14L3, "In the α-domain of the proteasome there are 19 leucine and 21 valine residues. Despite its high molecular weight, cross peaks were present for 92% of the methyl resonances in the 3D HMBC-HMQC experiment (68 detected out of a possible 74)." What is the reason for the discrepancy between the number of residues (40) and the possible number of resonances (74)?*

We thank the reviewer for pointing this inaccuracy out. We had previously used '74' on the basis of the previously known assignment. We have now rephrased and been explicit about the numbers:

In previous studies, 74 leucine and valine methyl-resonances were assigned out of possible 80 methyl-resonances[7]. Despite its high molecular weight and slow tumbling, cross peaks with S/N > 3 were observed for 80% of the methyl resonances in the 3D-HMBC-HMQC experiment (64 out of 80). Overall, 87.5% of the methyl groups in valine and leucine (35 residues out of 40) side chains were correctly paired.

4. *Intra-residue resonances could be linked for the 114 residues out of 116 in MSG, and the 35 residues out of 37 in half-proteasome. How about the other unlinked residues? Could the authors detect the cross-peaks for these residues? Were the transverse relaxation rates too larger?*

In case of MSG, as mentioned on page 11, the 3D-HMBC-HMQC spectrum confirms all nine tentative assignments made previously by a backbone dependent assignment and also permits the assignment of the two missing methyl groups, one for L174 (^{13}C=25.70 ppm and ^{1}H=0.95 ppm) and one for V620 (^{13}C=21.45 ppm and ^{1}H=0.82 ppm). Apart from these two new assignments, we were not able to detect cross peaks for the other unassigned methyl groups of MSG or the half-proteasome.

5. *What do the authors think about the applicability of the non-uniform sampling (NUS) to the 3D HMBC-HMQC spectra, which exhibit limited numbers of resonances?*

In the current study we chose to record the fully and uniformly sampled spectra. This allowed us to accurately judge the performance of the method and also accurately determine the noise. However, in future applications, we would expect that NUS should be fully applicable to 3D-HMBC-HMQC spectra due to the limited number of peaks present, as the reviewer points out.

6. *Even in the case of the assignments of methyl resonances with mutational analyses, the 3D HMBC-HMQC spectra would be useful to confirm the assignments, because mutations often affect the chemical shifts of neighboring residues. The authors may discuss this point.*

We agree with the reviewer that the HMBC-HMQC experiment could also be useful when the assignment is carried out using a mutational approach. To highlight this, we have now added the following in the conclusion:

Moreover, when methyl resonances are assigned using a mutational approach[7,8], the 3D-HMBC-HMQC could also provide valuable insight to confirm the assignments. Finally, …
